# Architecture Design and Interface Engineering of Self-assembly VS_4_/rGO Heterostructures for Ultrathin Absorbent

**DOI:** 10.1007/s40820-022-00809-5

**Published:** 2022-02-25

**Authors:** Qi Li, Xuan Zhao, Zheng Zhang, Xiaochen Xun, Bin Zhao, Liangxu Xu, Zhuo Kang, Qingliang Liao, Yue Zhang

**Affiliations:** Academy for Advanced Interdisciplinary Science and Technology, Beijing Advanced Innovation Center for Materials Genome Engineering, Beijing Key Laboratory for Advanced Energy Materials and Technologies, School of Materials Science and Engineering, University of Science and Technology Beijing, Beijing, 100083 People’s Republic of China

**Keywords:** Architecture design, Interface, Self-assembly, Microwave absorption

## Abstract

The employment of microwave absorbents is highly desirable to address the increasing threats of electromagnetic pollution. Importantly, developing ultrathin absorbent is acknowledged as a linchpin in the design of lightweight and flexible electronic devices, but there are remaining unprecedented challenges. Herein, the self-assembly VS_4_/rGO heterostructure is constructed to be engineered as ultrathin microwave absorbent through the strategies of architecture design and interface engineering. The microarchitecture and heterointerface of VS_4_/rGO heterostructure can be regulated by the generation of VS_4_ nanorods anchored on rGO, which can effectively modulate the impedance matching and attenuation constant. The maximum reflection loss of 2VS_4_/rGO40 heterostructure can reach − 43.5 dB at 14 GHz with the impedance matching and attenuation constant approaching 0.98 and 187, respectively. The effective absorption bandwidth of 4.8 GHz can be achieved with an ultrathin thickness of 1.4 mm. The far-reaching comprehension of the heterointerface on microwave absorption performance is explicitly unveiled by experimental results and theoretical calculations. Microarchitecture and heterointerface synergistically inspire multi-dimensional advantages to enhance dipole polarization, interfacial polarization, and multiple reflections and scatterings of microwaves. Overall, the strategies of architecture design and interface engineering pave the way for achieving ultrathin and enhanced microwave absorption materials.

**Supplementary Information:**

The online version contains supplementary material available at 10.1007/s40820-022-00809-5.

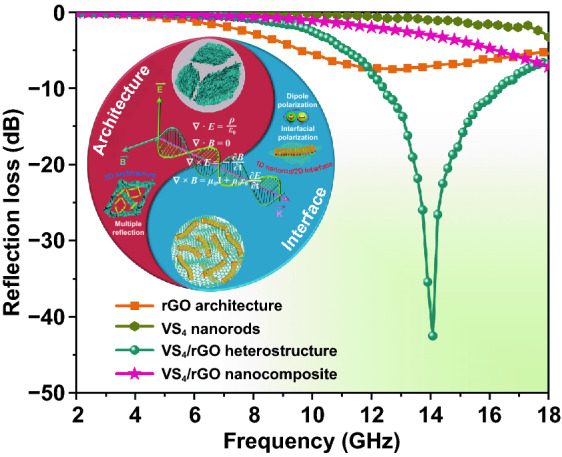

## Article Highlights

- The self-assembly VS_4_/rGO heterostructure is constructed to be engineered as ultrathin microwave absorbent through the strategies of architecture design and interface engineering.
- Microarchitecture and heterointerface synergistically inspire multi-dimensional advantages to enhance microwave absorption performance.
- The effective absorption bandwidth of 4.8 GHz can be achieved with an ultrathin thickness of 1.4 mm.

## Introduction

With the advent of the 5G era, widespread applications of communication technologies and electronic devices have caused serious electromagnetic (EM) interference [1, 2]. The ever-increasing demands for the elimination of EM radiation hazards have spurred significant concerns to develop microwave absorption materials [3, 4]. Recently, amounts of traditional materials, such as ferrites [5], metallic magnets [6], and dielectric ceramics have been widely employed as microwave absorbents [7]. However, the inherent defects such as larger thickness, high density, and narrow effective absorption bandwidth (EAB) seriously hinder their extensive applications, which is far from fulfilling the ultrathin and enhanced goals of microwave absorbent. Additionally, the boom of flexible and miniaturized smart electronic devices is eagerly demanding ultrathin and enhanced microwave absorbent [8]. Thus, it is highly desired but remains a gigantic challenge to achieve high-performance microwave absorbent.

Currently, considerable attention has been focused on the design of high-performance microwave absorbents to tackle the issues of ultra-thick thickness, high density, and imperfect absorption intensity and bandwidth [9, 10]. Carbon nanostructures, including graphene [11], carbon nanotubes [12], fullerene [13], and so on, demonstrate great excellences to be designed as ultrathin and enhanced microwave absorbent because of their large specific surface area, remarkable thermal and electric conductivities, and exceptional dielectric properties [14]. Reduced oxide graphene (rGO) as a widely studied two-dimensional (2D) material possesses residual oxygen-containing functional groups and defects that can be acted as attachment sites or dipole polarization centers [15–17]. The pristine 2D rGO nanosheet has great potential to be designed as an ultrathin microwave absorbent but exhibits marginal microwave attenuation capability, which cannot bridge the gap of achieving enhanced microwave absorption. In order to cope with this issue, rGO microarchitectures with multiple winkles and microporous cavities are tailored and widely applied to promote microwave attenuation capability in virtue of the multiple reflections and scatterings of incident microwaves [18–20]. The elaborate rGO microarchitecture fully maintains the superior privileges of rGO by evading excessive stacks or agglomerations, which can greatly contribute to pursuing the ultrathin microwave absorbent [21]. For example, Pan et al. prepared hierarchical multi-dimensional carbon-based aerogels. The graphene layers were uniformly intercalated by three-dimensional (3D) helical carbon nanocoils, endowing the abundant microporous structure and better dielectric properties. The hierarchical multi-dimensional carbon-based aerogels showed the maximum reflection loss (RL) of − 55.1 dB and EAB of 5.6 GHz [18]. Gao et al. designed graphene microflowers with highly microporous architecture. The maximum RL and EAB of graphene microflowers were up to − 42.9 dB and 5.59 GHz, respectively, showing significant increment compared with the stacked graphene, which is primarily due to the ascendancy microarchitecture [22]. Dielectric losses, including dipole and interfacial polarization relaxations, are predominant in rGO microwave absorbent, whereas the pristine rGO possessing single dipole polarization relaxation is difficult to obtain multiple dielectric losses. Therefore, introducing a heterointerface to stimulate interfacial polarization relaxation of rGO-based absorbent is another smooth road to enhance microwave absorption performance. For instance, Ji et al. anchored metal oxide granular film on graphene with large surface area and high charge carrier concentrations to construct heterointerface. The prepared graphene/metal oxide exhibited the EAB of 7.0 GHz with a thickness of 2 mm because of the strong interfacial polarization relaxation [23]. He et al. successfully developed rGO/MoS_2_ nanosheets heterostructure. The prepared rGO/MoS_2_ nanosheets heterostructure possessing multiple conductive networks and heterointerfaces demonstrated the EAB of 5.7 GHz and maximum RL of − 60 dB with a thickness of 2.5 mm [24]. Thence, the inspired interfacial polarization relaxation usually can endow rGO-based absorbent with enhanced microwave attenuation capability. VS_4_ with a narrow bandgap of ≈1.0 eV stands out from the transition metal sulfides due to its Peierls distortions, good dielectric characteristics, and environmental benignities. Moreover, the unique chain-like VS_4_ nanostructure formed by van der Waals force is easy to anchor on rGO to generate heterointerface (Fig. S1) [25]. Accordingly, motivated by the unparalleled merits of rGO microarchitecture and VS_4_ nanostructure, the synergy of microarchitecture and heterointerface may provide exceptional feasibilities to achieve enhanced VS_4_/rGO microwave absorption materials.

Herein, architecture design and interface engineering strategies are implemented to prepare self-assembly VS_4_/rGO heterostructure as ultrathin microwave absorbent. VS_4_ nanorods are tightly and evenly anchored on rGO, generating abundant microarchitectures and rich heterointerfaces. The synergy of microarchitecture and heterointerface effectively modulates the impedance matching and attenuation constant of VS_4_/rGO heterostructure, thus achieving the ultrathin absorbent with enhanced microwave absorption performance. The maximum RL of 2VS_4_/rGO40 heterostructure can reach as strong as − 43.5 dB at 14 GHz and 1.5 mm with the impedance matching and attenuation constant approaching 0.98 and 187, respectively. Furthermore, the EAB of 4.8 GHz can be achieved with an ultrathin thickness of 1.4 mm. More importantly, architecture design and interface engineering significantly contribute to motivating the multi-dimensional advantages of VS_4_/rGO heterostructure. (i) The anisotropic one-dimensional (1D) VS_4_ nanorods with high aspect ratio greatly facilitate dipole and interfacial polarization relaxations. (ii) 2D rGO nanosheets with VS_4_ nanorods attached can effectively modulate the impedance matching and boost interfacial polarization. (iii) 3D reticulum-like microporous architecture of VS_4_/rGO heterostructure induces multiple reflections and scatterings of incident microwaves. Moreover, combining with density functional theory calculation, the effects of VS_4_/rGO heterointerface for polarization relaxation are also further investigated, inducing reinforcement in microwave absorption performance. Overall, the synergy of architecture design and interface engineering for the construction of self-assembly VS_4_/rGO heterostructure can extend the development of ultrathin microwave absorbent.

## Experimental Section

### Synthesis of rGO Microarchitecture

Graphene oxide (GO) was prepared by the modified Hummers method from graphite powder. rGO architecture was synthesized by facile hydrothermal and freeze-dying methods. Typically, 80 mg of GO powder was uniformly dispersed in 60 mL deionized water by sonication of 90 min. Then, the prepared uniformly homogeneous GO dispersion was transferred into a Teflon-lined stainless autoclave and heated to 180 °C for 10 h. After cooling to room temperature and freezing in the refrigerator at − 20 °C, the frozen hydrogel was put into a vacuum freeze dryer to remove ice and acquire rGO architecture.

### Synthesis of VS_4_/rGO Heterostructure

Typically, the VS_4_/rGO heterostructures were successfully performed by a facile one-step hydrothermal method. Initially, 5 mmol of Na_3_VO_4_ and 25 mmol of CH_3_CSNH_2_ were put into 60 mL dispersion containing 40 mg of GO under constant magnetic stirring until formed a homogeneous solution at 60 °C. Then, the mixed solution was transferred into 100 mL of Teflon-lined stainless autoclave and heated to 160 °C for 24 h. After cooling to room temperature, the product was collected and washed with deionized water three times and placed in the refrigerator at − 20 °C for 12 h. Subsequently, the frozen product was performed to remove the water of VS_4_/rGO heterostructures via a freeze-drying method. As Table S1 shows, the VS_4_/rGO heterostructures with different content of VS_4_ nanorods and rGO marked as 1VS_4_/rGO40, 3VS_4_/rGO40, 2VS_4_/rGO20, and 2VS_4_/rGO60 were prepared by the same method.

### Synthesis of VS_4_/rGO Nanocomposite

VS_4_ nanorods were prepared via a hydrothermal method. In a typical process, 5 mmol of Na_3_VO_4_ and 25 mmol of CH_3_CSNH_2_ were dissolved in 60 mL of deionized water to obtain the mixed solution, and then 1 mol L^−1^ NaOH aqueous solution was added to manipulate the pH of 12. After magnetic stirring of 0.5 h at 60 °C, the solution was transferred into 100 mL of Teflon-lined stainless autoclave and heated to 160 °C for 24 h. After cooling to room temperature, the product was collected and washed with deionized water three times. Then, the purified product was dried in a vacuum oven at 60 °C for 12 h to obtain the pristine VS_4_ nanorods.

VS_4_/rGO nanocomposite was prepared by the ultrasonic dispersion method. Detailly, the VS_4_ nanorods and rGO were dispersed into the absolute ethanol under ultrasound condition, and the molar ratio of VS_4_ nanorods and rGO was about 3:1. Then, the prepared VS_4_/rGO nanocomposite was obtained by centrifugal separation and vacuum drying method.

### Characterization and EM Parameters Measurement

The morphology and microstructure of the prepared VS_4_/rGO heterostructures were characterized by field emission scanning electron microscope (FESEM; FEI, Quanta3D FEG) and transmission electron microscope (TEM; TECNI G2 F20). The crystal structures were measured by X-ray diffraction (XRD; Rigaku DMAX-RB). Raman spectra were obtained by Jobin–Yvon Raman microprobe (JY-HR800) under 532 nm laser excitation. Chemical compositions and valence states were analyzed by X-ray photoelectron spectrometer (XPS; Thermo Fisher ESCALab250). Thermo-gravimetric analysis (TGA) was conducted in dry air by using a TA Q-600. The nitrogen adsorption/desorption experiments were performed in Micromeritics ASAP2020. The specific surface area was obtained by using Brunauer–Emmett–Teller (BET) analysis. Pore size distributions were assessed by using the Barrett–Joyner–Halenda (BJH) method.

The EM parameters, including permittivity and permeability, were measured by a vector network analyzer system (HP722ES) in the frequency range of 2–18 GHz. The VS_4_/rGO heterostructures were uniformly dispersed in paraffin with filler loadings of 20%, 30%, and 40%. Then, the as-prepared mixture was pressed into a toroidal-shaped specimen with an inner diameter of 3.04 mm, an outer diameter of 7.0 mm, and a thickness of 2.0 mm. The EM parameter measurements of VS_4_ nanorods, rGO architecture, and VS_4_/rGO nanocomposite were like VS_4_/rGO heterostructure. The complex permittivity and permeability of the as-prepared samples were further to be measured.

### DFT Calculation

The Vienna Ab-initio Simulation Package (VASP) software was used to simulate computation based on density functional theory (DFT). The interaction between ions and electrons was described by the projector-augmented wave (PAW) method. The Generalized Gradient Approximation (GGA) with the Perdew Burke Ernzerhof (PBE) function was employed as the exchange associated functions.

## Results and Discussion

**Fig. 1:**
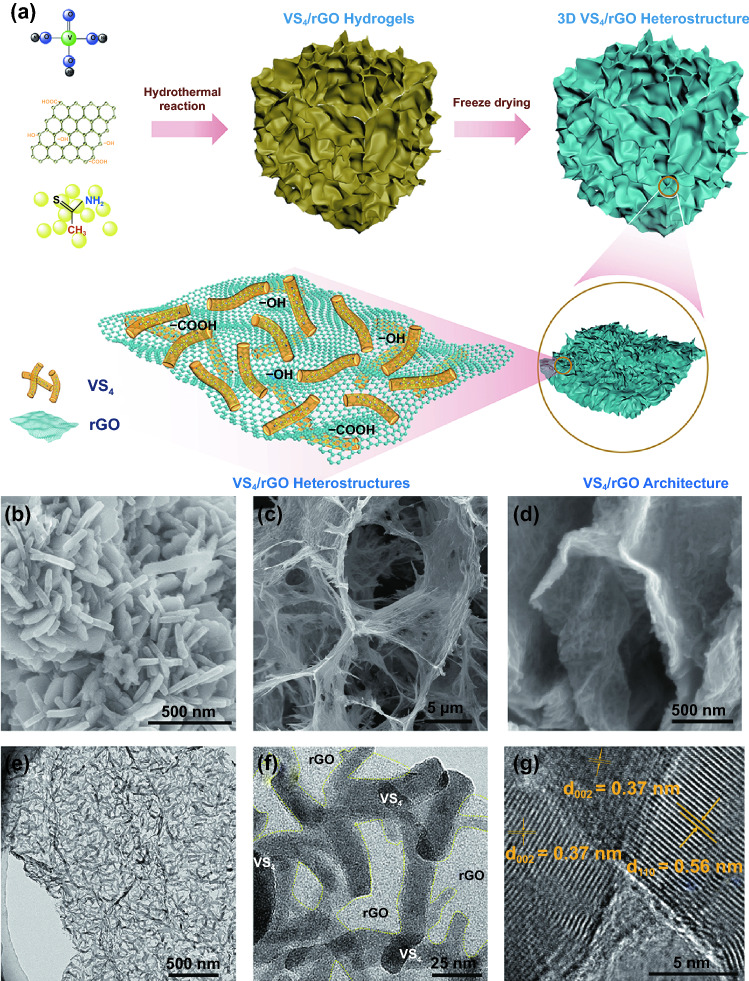
**a** Schematic illustration of the synthesis processes for self-assembly VS_4_/rGO heterostructure. SEM image of **b** VS_4_ nanorods, **c** rGO architecture, and **d** VS_4_/rGO heterostructure. **e, f** TEM images of VS_4_/rGO heterostructure with different magnification. **g** HRTEM images of VS_4_ nanorods showing the d-spacing of 0.56 nm in the (110) plane and rGO showing the d-spacing of 0.37 nm in (0 0 2) plane

Figure 1a schematically illustrates the synthetic process of self-assembly VS_4_/rGO heterostructure. The VS_4_/rGO heterostructure with abundant microarchitectures and rich heterointerfaces was fabricated through hydrothermal and freeze-dying methods. Similarly, the pristine VS_4_ nanorods were fabricated without rGO by the same hydrothermal reaction. Meanwhile, Fig. 1 also shows a series of SEM and TEM images of the VS_4_ nanorods and self-assembly VS_4_/rGO heterostructure. Figure 1b reveals that the VS_4_ nanorods are seriously agglomerated due to the absence of a growing template. In Fig. 1c, the rGO architecture demonstrates reticulum-like microarchitecture with lots of randomly distributed nano-walls formed by wrinkled nanosheets. Figures 1d and S2 exhibit that the self-assembly VS_4_/rGO heterostructure also possesses numerous wrinkles and microporous architecture originated from scrolling and folding of rGO. The crooked VS_4_ nanorods are evenly anchored on the surface of rGO as Fig. 1e elucidated. Furthermore, the heterointerfaces between VS_4_ nanorods and rGO are clearly shown in Fig. 1f, and the average lateral and longitudinal sizes of VS_4_ nanorods are about 20–40 and 100–300 nm, respectively. High-resolution transmission electron microscopy (HRTEM) image of VS_4_/rGO heterostructure with distinct lattice is exhibited in Fig. 1g. The interlayer spacings of 0.56 nm and 0.37 nm correspond to (1 1 0) plane of VS_4_ and (0 0 2) plane of rGO, respectively [25]. Therefore, the interface between VS_4_ and rGO can be clearly observed, but the distinct voids and gaps at boundaries do not appear, exhibiting the tight combination of VS_4_ nanorods and rGO.

**Fig. 2:**
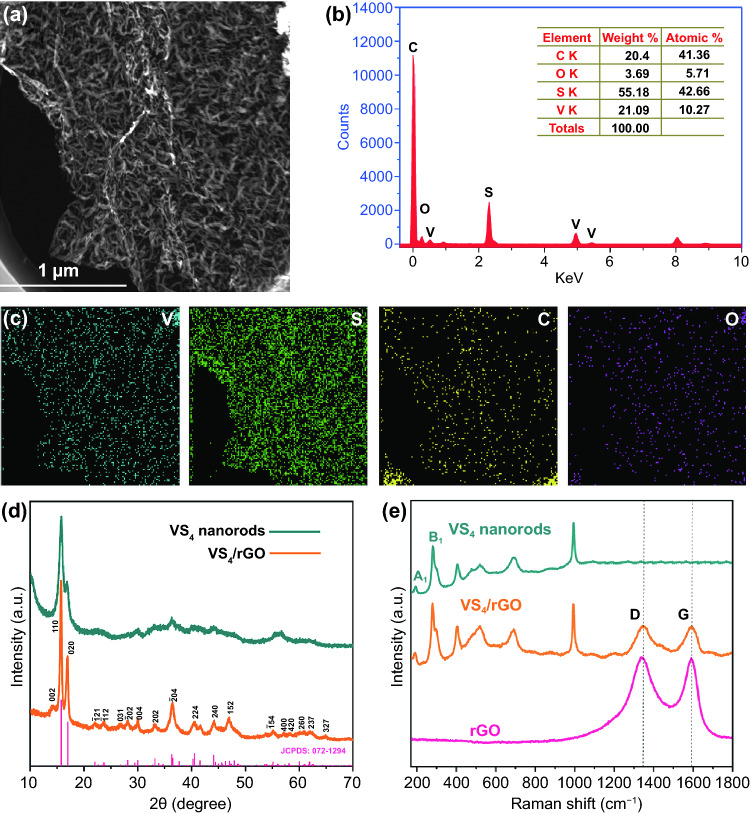
**a** TEM image of VS_4_/rGO heterostructure for the EDS mapping. **b** EDS spectra and the table of elemental composition. **c** TEM image corresponding to EDS elemental mappings: V, S, C and O. **d** XRD patterns of VS_4_ nanorods and VS_4_/rGO heterostructures. **e** Raman spectra of VS_4_ nanorods, rGO and VS_4_/rGO heterostructure

The elemental compositions of VS_4_/rGO heterostructure are further measured by energy dispersive spectroscopy (EDS). Figure 2a presents the VS_4_ nanorods are horizontally and evenly aligned on the rGO. Figure 2b shows that the elements of V, S, C, and O can be identified from the survey scan of EDS with the atomic proportions of 10.27, 42.66, 41.36, and 5.71 at%, respectively. The stoichiometric ratio of V and S atoms is around 1: 4, which proves the existence of VS_4_ [25]. Further confirmed by the elemental mapping of Fig. 2c, the four elements of V, S, C, and O are detected on the surface of VS_4_/rGO heterostructure. The C element is derived from rGO, and the O element mainly originates from the residual oxygen-containing functional groups of rGO [26, 27]. The crystalline phase of the prepared VS_4_/rGO heterostructure is revealed by X-ray diffraction (XRD). Figure 2d shows the two major peaks of 15.8° and 17.0° are assigned to the (1 1 0) and (0 2 0) planes of monoclinic VS_4_ (JCPDS No. 072–1294) [28]. Figure 2e demonstrates the Raman spectra of VS_4_ nanorods, rGO, and VS_4_/rGO heterostructure. The peaks located at 191.6 and 280 cm^−1^ are attributed to the stretching (A_1_) and bending (B_1_) modes of V-S chemical bands, respectively. The characteristic peaks located at 1342 and 1593 cm^−1^ correspond to the D and G peaks of rGO. In addition, the peak intensity ratio of D and G for VS_4_/rGO heterostructure (1.1) is higher than rGO (0.96), revealing the formation of plentiful defects and distortions caused by structural imperfection and heterointerface [29].

**Fig. 3:**
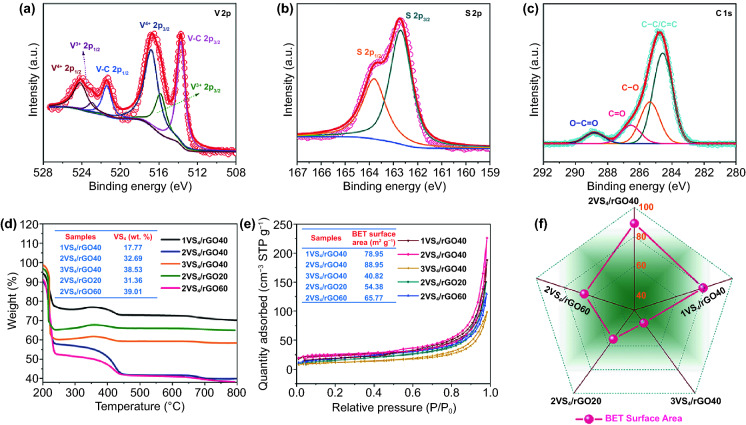
XPS spectra of VS_4_/rGO heterostructures: **a** V 2p, **b** S 2p, **c** C 1s. **d** TGA curves of VS_4_/rGO heterostructures. **e** Nitrogen adsorption–desorption isotherms. **f** Different BET surface areas of VS_4_/rGO heterostructures

The surface chemical state of VS_4_/rGO heterostructure is investigated by X-ray photoelectron spectroscopy (XPS) (Fig. S3). Figure 3a shows the high-resolution XPS spectrum of V 2p. The characteristic peaks of 516.5 and 524 eV are attributed to V 2p_3/2_ and V 2p_1/2_, corresponding to the V^4+^ of VS_4_ [30, 31]. The other two characteristic peaks of 513.9 and 521.8 eV derived from V-C chemical bonds can be clearly detected, confirming the strong bonding and abundant heterointerface between VS_4_ and rGO [32]. In contrast, the high-resolution XPS spectra of VS_4_ nanorods and VS_4_/rGO nanocomposite prepared by ultrasonic dispersion method are shown in Figs. S4 and S5, respectively. As shown in Fig. S5a, the high-resolution spectrum of V 2p lacks the V-C band peak, which is due to VS_4_ nanorods fail to be fabricated and anchored on rGO. Figure S5b-c exhibits that the high-resolution spectra of S 2*p* and C 1*s* are the same as the VS_4_/rGO heterostructure. Compared with VS_4_ nanorods and VS_4_/rGO nanocomposite, the V 2p_3/2_ of VS_4_/rGO heterostructure demonstrates a positive shift, indicating the electrons transfer from VS_4_ nanorods to rGO (Fig. S6). As Fig. 3b shown, the corresponding peaks located at 162.7 and 163.8 eV originating from the S_2_^2−^ ions can be indexed to S 2*p*_3/2_ and S 2*p*_1/2_, respectively [32]. Figure 3c shows the fitted C 1*s* peaks approximately locate at 284.7, 285.7, 286.7, and 288.6 eV, respectively. These C 1*s* peaks are originated in the presences of C–C/C=C in the aromatic ring and the residual oxygen-containing functional groups such as –OH and –COOH [33].

The VS_4_/rGO heterostructures are analyzed by thermo-gravimetric analysis (TGA) to evaluate their thermal stability and content. As Fig. 3d shows, the small weight loss of VS_4_/rGO heterostructures below 200 °C is attributed to the desorption of moisture. The large weight losses over 200–250 and 250–350 °C are related to the decomposition of VS_4_ nanorods and the oxidation reaction of sulfur compounds, respectively. The oxidation decomposition of rGO is observed at 370–500 °C. After 600 °C, the slight weight gain is caused by the further oxidation of vanadium oxide [33, 34]. The N_2_ adsorption–desorption isotherm is obtained to measure the surface area and pore size distribution of VS_4_/rGO heterostructure. Figure 3e shows that the isotherms of VS_4_/rGO heterostructures exhibit type-IV curve accompanied by weak hysteresis loop, thus implying the presence of numerous mesopores. In order to evaluate the microarchitecture and heterointerface of self-assembly VS_4_/rGO heterostructure, BET surface area and TGA result are combined to clarify their relationships. Firstly, the amount of GO (40 mg) remains unchanged to regulate the preparation of VS_4_ nanorods and modulate the VS_4_/rGO heterointerface. Generally, the VS_4_/rGO heterostructure with the higher content of VS_4_ nanorods causes the larger VS_4_/rGO heterointerface formed by the attachment of VS_4_ on rGO. The BET surface area usually can indicate the abundance of microarchitecture. The contents of VS_4_ nanorods for 1VS_4_/rGO40, 2VS_4_/rGO40, and 3VS_4_/rGO40 calculated by TGA results are about 17.75, 32.69, and 38.53 wt%, respectively. Correspondingly, the BET surface areas of 1VS_4_/rGO40, 2VS_4_/rGO40, and 3VS_4_/rGO40 measured by nitrogen adsorption–desorption isotherms can reach 78.95, 88.95, and 40.82 m^2^ g^−1^, respectively. 2VS_4_/rGO40 with the optimal 32.69 wt% of VS_4_ nanorods possesses the largest BET surface area of 88.78 m^2^ g^−1^, manifesting abundant microarchitectures and rich heterointerfaces. However, rGO cannot provide enough attachment sites for the excessive generation of VS_4_ nanorods, causing the serious agglomeration of VS_4_ nanorods. Agglomerated VS_4_ nanorods will wreck the inherent microarchitecture and further shrink the BET surface area. Wherefore, 3VS_4_/rGO40 with the highest 38.53 wt% of VS_4_ nanorods possesses the smallest BET surface area of 40.82 m^2^ g^−1^, leading to insufficient microarchitectures. 1VS_4_/rGO40 with the lowest 17.75 wt% of VS_4_ nanorods usually generates the minimal heterointerface but higher BET surface area derived from rGO. The BET surface area of 1VS_4_/rGO40 is smaller than 2VS_4_/rGO40, which is mainly due to the shortage of the support of VS_4_ nanorods. Secondly, the content of the prepared VS_4_ nanorods remains unchanged within a certain range to regulate the amount of GO. The contents of VS_4_ nanorods for 2VS_4_/rGO20 and 2VS_4_/rGO60 are up to 31.36 and 39.01 wt%, respectively. Correspondingly, the BET surface areas of 2VS_4_/rGO20 and 2VS_4_/rGO60 can reach 54.38 and 65.77 m^2^ g^−1^, respectively. Figure 3f clearly displays the differences on BET surface area of the fabricated VS_4_/rGO heterostructures. Generally, the optimized amount of GO can provide a suitable surface and active sites to anchor VS_4_ nanorods. On the contrary, the excessive amount of GO cannot make the specific content of VS_4_ nanorods evenly attach to its surface. The prepared VS_4_ nanorods are not connected to each other and cannot provide strong skeleton support for rGO microarchitecture. Therefore, the local collapse of the microarchitecture of 2VS_4_/rGO60 hinders the achievement of the largest BET surface area. 2VS_4_/rGO20 demonstrates a smaller BET surface area of 54.38 m^2^ g^−1^ than 2VS_4_/rGO40, which is mainly due to the less content of GO.

In general, combined with TGA results of VS_4_/rGO heterostructure, BET surface area can accurately reveal the relationship of microarchitecture and heterointerface. The pore size distributions of VS_4_/rGO heterostructures are calculated by Barrett–Joyner–Halenda (BJH) method. The pore size distributions of reticulum-like microporous VS_4_/rGO heterostructures are shown in Fig. S7. The mesopores and macropores existing in the VS_4_/rGO heterostructure are associated with the content of GO in the hydrothermal reaction process. Additionally, 2VS_4_/rGO40 with the largest heterointerface area demonstrates more reticulum-like micropores that provide plentiful microporous networks [32–34].

**Fig. 4:**
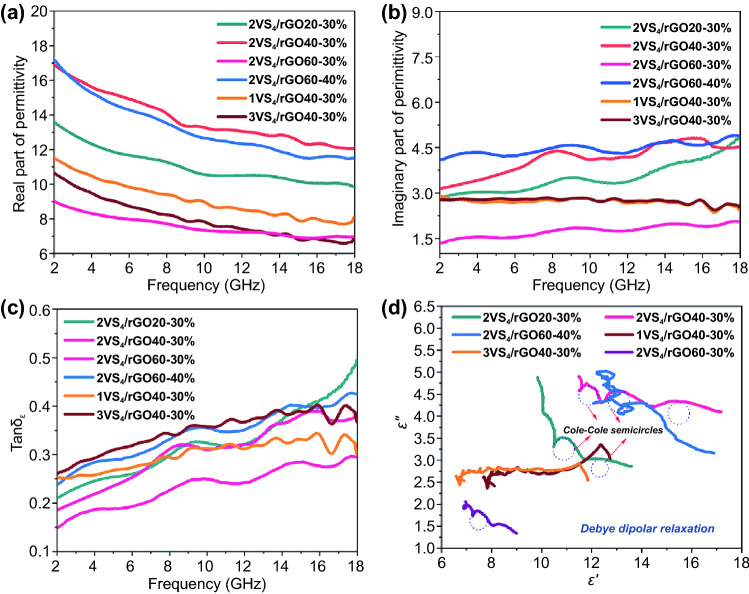
Permittivity and dielectric loss tangent of VS_4_/rGO heterostructures in the frequency range of 2–18 GHz: **a** ε′, **b** ε", **c** Tan δ_ε_. **d** Cole–Cole semicircles for VS_4_/rGO heterostructure

To evaluate the microwave absorption performance, the EM parameters, including complex permittivity (*ε*_*r*_ = *ε′-jε"*) and complex permeability (*μ*_*r*_ = *μ′-jμ″*), are measured by vector network analyzer system (HP722ES) in the frequency range of 2–18 GHz. According to the EM energy conversion principle, the real parts of complex permittivity (*ε′*) and permeability (*μ′*) are associated with electrical and magnetic energy storages, respectively. However, the imaginary parts of permittivity (*ε″*) and permeability (*μ″*) represent the dissipations of electric and magnetic energies, respectively [35]. As shown in Fig. 4a, the *ε′* of VS_4_/rGO heterostructures with the different filler loadings exhibit a downward tendency in the frequency range of 2–18 GHz. When the frequency of the microwave is increasing, the dipoles existing in VS_4_/rGO heterostructure cannot reorient themselves quickly enough to respond to the applied alternating EM field. Therefore, the complex permittivity starts to decrease and generates typical frequency dispersion behaviors.

When the filler loadings of 2VS_4_/rGO20, 2VS_4_/rGO40, and 2VS_4_/rGO60 are fixed at 30 wt%, the *ε′* of 2VS_4_/rGO40-30% demonstrates a decreasing tendency from 16.91 to 12.0, and the values of *ε′* for 2VS_4_/rGO20-30% and 2VS_4_/rGO60-30% all display decreased trends. The *ε′* value of 2VS_4_/rGO60-40% is higher than 2VS_4_/rGO60-30%, which is mainly due to the increase of dielectric media. The values of *ε′* for 1VS_4_/rGO40 and 3VS_4_/rGO40 maintain a relatively lower level. Figure 4b demonstrates the changing trend of *ε″* is different from *ε′*. In general, the values of *ε″* increase with the filler loading of VS_4_/rGO heterostructures, which is related to the presence of abundant microarchitectures and heterointerfaces. Based on the analysis of TGA results and BET surface area, 2VS_4_/rGO40 possesses abundant microarchitectures and rich heterointerfaces, demonstrating the highest ε″ than other samples with the filler loading of 30 wt%. The *ε″* of 2VS_4_/rGO40 is over 3.0 in the frequency range of 2–18 GHz, indicating the great EM energy attenuation ability. The other samples exhibit lower values of *ε″*, which are mainly due to the poor microarchitecture and insufficient heterointerface. Additionally, the multiple dielectric relaxation peaks exhibiting in the *ε″- f* curves are derived from nonlinear resonant behaviors of VS_4_/rGO heterointerfaces [36, 37]. Correspondingly, 2VS_4_/rGO40 demonstrates obvious and corrugated resonance peaks in the *ε″-f* curves of 7–11 GHz and 13–16 GHz, further manifesting the promotion of abundant microarchitectures and rich heterointerfaces for dielectric loss.

In order to evaluate the dielectric loss capability, the values of tanδ_ε_ (*tanδ*_*ε*_ = *ε″/ε′*) are shown in Fig. 4c. The curves of tanδ_ε_ display several fluctuations in the frequency range of 8–18 GHz, which are mainly due to the orientations of induced dipoles. Additionally, the dipole moments cannot match up with the alternating EM fields in high-frequency range. Furthermore, the weak magnetic properties of VS_4_/rGO heterostructure cause the values of *μ′* and *μ″* to fluctuate around 1 and 0, respectively. Therefore, the calculated tanδ_μ_ (*tanδ*_*μ*_ = *μ″/μ′*) of VS_4_/rGO heterostructure is closed to 0 (Fig. S8). According to Debye relaxation theory, the *ε*_*r*_ can be indicated as Eq. 1 [38]:

1 $${\varepsilon }_{r}={\varepsilon }_{\infty }+\frac{{\varepsilon }_{s}-{\varepsilon }_{\infty }}{1+i2\pi f\tau }={\varepsilon }^{^{\prime}}\left(f\right)+i{\varepsilon }^{^{\prime\prime} }\left(f\right)$$

where *f* represents the frequency, *τ* is the polarization relaxation time, *ε*_*r*_ and *ε*_*∞*_ represent the stationary permittivity and the optical dielectric constant, respectively. Meanwhile, the *ε′* and *ε″* can be further demonstrated as Eqs. 2 and 3:

2 $$\varepsilon {^{\prime}} = {\varepsilon }_{\infty }+ \frac{{\varepsilon }_{s}-{\varepsilon }_{\infty }}{1+{(2\pi f)}^{2}{\tau }^{2}}$$

3 $$\varepsilon {^{\prime}}{^{\prime}} = \frac{2\pi f\tau ({\varepsilon }_{s}-{\varepsilon }_{\infty })}{1+{(2\pi f)}^{2}{\tau }^{2}}$$

Based on the aforementioned Eqs. 1–3, the relationship between *ε′* and *ε″* can be clearly expressed as Eq. 4:

4 $${(\varepsilon {^{\prime}}-{\varepsilon }_{\infty })}^{2} + {(\varepsilon {^{\prime}}{^{\prime}})}^{2} = {({\varepsilon }_{s}-{\varepsilon }_{\infty })}^{2}$$

According to Eq. 4, the curve of *ε′* versus *ε″* exhibits a single semicircle denoted as Cole–Cole semicircle, which illustrates the process of Debye dipolar relaxation. Generally, the dielectric loss also can be obviously represented by Debye dipolar relaxation [39]. The curves of *ε′* versus *ε″* for VS_4_/rGO heterostructures are shown in Fig. 4d, in which the presence of semicircles demonstrates the multiple dielectric relaxation losses.

In order to evaluate the microwave absorption performance, the RL of VS_4_/rGO heterostructure usually can be calculated by the measured complex permittivity and permeability. The RL can be evaluated by Eqs. 5 and 6 based on the transmission line theory [40]:

5 $$Z_{{{\text{in}}}} = Z_{0} \left( {\mu _{r} /\varepsilon _{r} } \right)^{{1/2}} \tanh \left[ {j\left( {2\pi fd\left( {\mu _{r} \varepsilon _{r} } \right)^{{1/2}} /c} \right)} \right]$$

6 $$\mathrm{RL}=20\mathrm{log}\left|\frac{({Z}_{\text{in}}-{Z}_{0})}{({Z}_{\text{in}}+{Z}_{0})}\right|$$

**Fig. 5:**
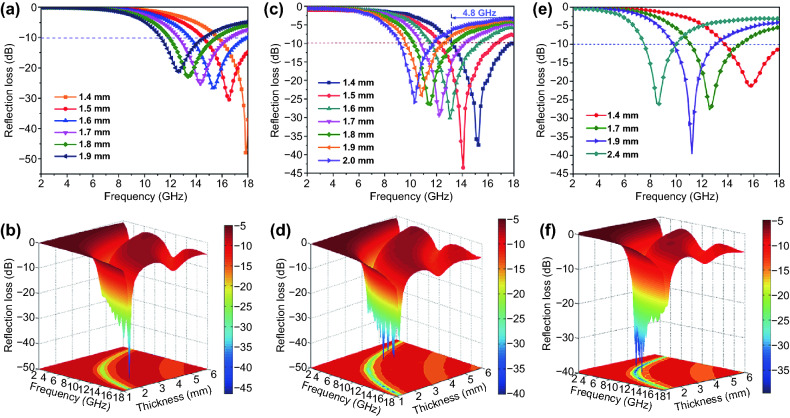
RL curves and 3D presentations of VS_4_/rGO heterostructures at the thicknesses of 1 to 6 mm in the frequency range of 2–18 GHz: **a, b** 2VS_4_/rGO20-30%; **c, d** 2VS_4_/rGO40-30%; **e, f** 2VS_4_/rGO60-40%

*Z*_*0*_ and *Z*_in_ represent the characteristic impedance of free space and the input impedance of absorbent, respectively. *f* is the frequency of the incident microwave; *t* is the thickness of the absorbent layer; *c* is the velocity of light. To reveal the influence of the frequency and thickness of absorbent, Fig. 5 outlines the RL curves and 3D presentations of the calculated RL of VS_4_/rGO heterostructures within the thickness of 1–6 mm and in the frequency range of 2–18 GHz.

The microwave absorption performance of VS_4_/rGO heterostructure can be modulated by manipulating the thickness and filler loading. The effective microwave absorption performance means that 90% of incident microwaves are attenuated, corresponding to the value of RL exceeding − 10 dB. Figure 5a-b illustrates that the maximum RL of − 48.4 dB for 2VS_4_/rGO20-30% can be achieved at 17.8 GHz with a thickness of 1.4 mm. Figure 5c-d shows that the EAB of 2VS_4_/rGO40-30% is up to 4.8 GHz (13.2–18 GHz) with a thickness of 1.4 mm, and the maximum RL can reach as strong as − 37.4 dB. Under the same thickness, the EAB of 4.8 GHz for VS_4_/rGO heterostructure is larger than that of other graphene-based microwave absorbents. Therefore, the VS_4_/rGO heterostructure has great potential in the design of ultrathin absorbents. The microwave absorption performances of 1VS_4_/rGO40-30% and 3VS_4_/rGO40-30% are shown in Fig. S9. The maximum RLs of 1VS_4_/rGO40-30% and 3VS_4_/rGO40-30% all reach − 25 dB, and their EABs are very narrow with a thickness of 1.4 mm. Figure 5e-f demonstrates that the EAB of 2VS_4_/rGO60-40% can reach as strong as 4.25 GHz with a thickness of 1.4 mm, and its maximum RL is up to − 39.5 dB at 11.2 GHz with a thickness of 1.9 mm. The microwave absorption performances of other VS_4_/rGO heterostructures with different filler loadings are demonstrated in Figs. S10 and S11.

**Fig. 6:**
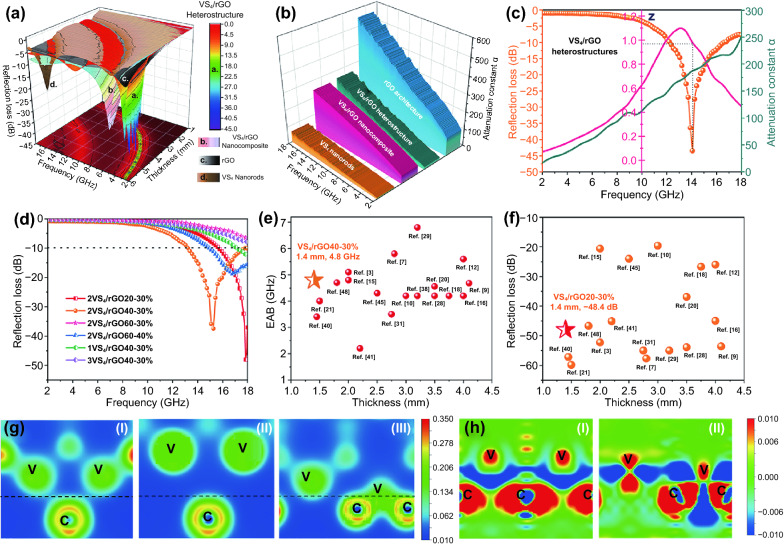
**a** 3D RL presentations of rGO, VS_4_ nanorods, VS_4_/rGO nanocomposite, and VS_4_/rGO heterostructure (2VS_4_/rGO40-30%). **b** Attenuation constant α of rGO, VS_4_ nanorods, VS_4_/rGO nanocomposite, and VS_4_/rGO heterostructures (2VS_4_/rGO40-30%). **c** Frequency dependence of RL, attenuation constant α and |Z_in_/Z_0_| at 1.5 mm for 2VS_4_/rGO40-30%. **d** RL of different VS_4_/rGO heterostructures at 1.4 mm. Comparison of some typical graphene-based absorbents for **e** EAB and **f** RL_max_ at different thickness. **g** Charge density of the interfaces (I) VS_4_(-2 0 4)/rGO (1 0 0) interface, (II) VS_4_(0 2 0)/rGO (1 0 0) interface, (III) VS_4_(1 1 0)/rGO (1 0 0) interface. **h** Charge density difference of (I) VS_4_(-2 0 4)/rGO (1 0 0) and (II) VS_4_(1 1 0)/rGO (1 0 0) interface

Figure 6a demonstrates the 3D RL presentations of rGO, VS_4_ nanorods, VS_4_/rGO nanocomposite, and VS_4_/rGO heterostructure (2VS_4_/rGO40-30%). Specifically, the maximum RLs of rGO architecture and VS_4_ nanorods are about − 7.5 and − 3.2 dB with a thickness of 1.5 mm, respectively. However, the maximum RL of 2VS_4_/rGO40-30% is up to − 43.5 dB at 14 GHz with a thickness of 1.5 mm, overwhelmingly outperforming the microwave absorption performances of rGO architecture and VS_4_ nanorods. In contrast, the VS_4_/rGO nanocomposite prepared by the ultrasonic dispersion method shows the weak RL of − 6.9 dB with a thickness of 1.5 mm (Fig. S11a). The self-assembly VS_4_/rGO heterostructure holds the intimate contact heterointerface that is proved by the previous characterizations of TEM and XPS. However, the pristine VS_4_ nanorods and rGO architecture are short of heterointerfaces, and the absence of interfacial polarization relaxation fails to promote microwave attenuation. So, it can be concluded that the VS_4_/rGO heterointerface benefits the enhancement of microwave absorption performance. Compared with the VS_4_/rGO nanocomposite, the VS_4_/rGO heterostructure with rich microporous architectures can greatly improve the multiple reflections and scatterings of microwaves, which exponentially boost microwave attenuation. The *ε*′, *ε*″, and tan*δ*_ε_ of VS_4_ nanorods, rGO architecture, VS_4_/rGO nanocomposite, and VS_4_/rGO heterostructure are shown in Fig. S11. As Fig. S11b-c shown, rGO architecture demonstrates the highest *ε*′ and *ε*″. Undoubtedly, rGO possessing a significant dielectric property endows the self-assembly VS_4_/rGO heterostructure with greater permittivity. In contrast, VS_4_/rGO nanocomposite prepared by ultrasonic dispersion cannot effectively inspire the excellent dielectric property of rGO. The pristine VS_4_ nanorods have limited dielectric properties because of their inherited attributes. The rGO architecture also has the highest tan*δ*_ε_, further manifesting its excellent dielectric loss. VS_4_/rGO nanocomposite displays a higher value of tan*δ*_ε_ than the self-assembly VS_4_/rGO heterostructure, which is mainly originated in the independent rGO (Fig. S11d). The tanδ_ε_ of self-assembly VS_4_/rGO heterostructure is also higher than the agglomerated VS_4_ nanorods, proving that the introduction of rGO plays a great role in boosting dielectric loss. Additionally, the permeabilities of VS_4_ nanorods, rGO architecture, VS_4_/rGO nanocomposite, and VS_4_/rGO heterostructure are negligible due to the weak magnetic performances of VS_4_ and rGO. The microwave absorption performances of rGO, VS_4_ nanorods, and VS_4_/rGO nanocomposite with different thicknesses are shown in Fig. S12.

Generally, the excellent microwave absorption performance not only comes from the higher attenuation capability but also attributes to the well-matched impedance matching. The attenuation capability of absorbent is evaluated by the concept of attenuation constant *α*, which can be calculated by Eq. 7 [41]:

7 $$\alpha =\frac{\sqrt{2}}{c}\pi f\times \sqrt{\left({\mu }^{^{\prime\prime} }{\varepsilon }^{^{\prime\prime} }-{\mu }^{^{\prime}}{\varepsilon }^{^{\prime}}\right)+\sqrt{{\left({\mu }^{^{\prime\prime} }{\varepsilon }^{^{\prime\prime} }-{\mu }^{^{\prime}}{\varepsilon }^{^{\prime}}\right)}^{2}+{\left({\mu }^{^{\prime}}{\varepsilon }^{^{\prime\prime} }+{\mu }^{^{\prime\prime} }{\varepsilon }^{^{\prime}}\right)}^{2}}}$$

Figure 6b illustrates that rGO possesses the highest attenuation constant due to its excellent dielectric loss and conductive loss. VS_4_/rGO heterostructure and VS_4_/rGO nanocomposite exhibit a similar value of attenuation constant, proving the combination of VS_4_ and rGO is good for achieving higher attenuation constant. The pristine VS_4_ nanorods demonstrate the weak attenuation constant, which is negative to microwave absorption. Among them, the VS_4_/rGO heterostructure exhibits the best microwave absorption performance, which is mainly due to its well-matched impedance matching. The frequency dependences of impedance matching, attenuation constant, and RL for 2VS_4_/rGO40-30% with a thickness of 1.5 mm are shown in Fig. 6c. According to Eq. 5, the value of *Z* =*|Z*_in_*/Z*_*0*_*|* closed to 1 means that all microwaves propagate into the interior of absorbent without reflections [42]. Generally, the attenuation constant can attain a larger value in the high-frequency range. However, the maximum RL cannot be obtained after the 16 GHz due to the value of *Z* is far away 1. The lower value of impedance matching is attributed to the strong interfacial reflection of microwave. Taking impedance matching and attenuation constant into account, the maximum RL of 2VS_4_/rGO40-30% reaches − 43.5 dB in 14 GHz with the impedance matching approaching 1. The frequency dependences of impedance matching, attenuation constant, and RL for VS_4_ nanorods, rGO architecture, and VS_4_/rGO nanocomposite at the matching thickness of 1.5 mm are shown in Fig. S13. As Fig. S13a shows, rGO architecture demonstrates the higher attenuation constant and poor impedance matching, leading to weak microwave absorption performance. Figure S13b shows that VS_4_ nanorods possess the better impedance matching and lower attenuation constant, demonstrating limited microwave absorption performance. Figure S13c shows that the VS_4_/rGO nanocomposite prepared by the ultrasonic dispersion method also presents weak microwave absorption performance with a thickness of 1.5 mm, and the mismatched impedance matching significantly weakens microwave absorption performance even though the attenuation constant is at high level [43].

Except for the existence of heterointerface, the richness of heterointerface also exerts great influences on the microwave absorption performance. According to the above analysis of BET surface area and TGA results, the heterointerface and microarchitecture of VS_4_/rGO heterostructure can be modulated by the self-assembly generation of VS_4_ nanorods anchored on rGO. Figure 6d shows the microwave absorption performance of different VS_4_/rGO heterostructures with a thickness of 1.4 mm in the frequency range of 2–18 GHz. 2VS_4_/rGO40 with the optimal mass ratio of VS_4_ and rGO as well as the largest BET surface area of 88.95 m^2^ g^−1^ possesses the EAB of 4.8 GHz with an ultrathin thickness of 1.4 mm. Without a doubt, the EABs of 1VS_4_/rGO40, 3VS_4_/rGO40, 2VS_4_/rGO20, and 2VS_4_/rGO60 are weaker than 2VS_4_/rGO40, which chiefly derives from their insufficient microarchitectures and heterointerfaces. The comparisons of microwave absorption performance between VS_4_/rGO heterostructure and other graphene-based absorbents are shown in Fig. 6e-f as well as Table 1. As the consequence, the EAB and RL of VS_4_/rGO heterostructure outperform most of the graphene-based absorbents with a thickness of 1.4 mm.

**Table 1:** Typical graphene-based heterostructure and their microwave absorption performance

Samples	Thickness (mm)	RL_max_ (dB)	< -10 dB (GHz)	Refs.
CMT@CNT/Co	2.0	− 52.3	5.1	[3]
CFO/rGO	2.8	− 57.7	5.8	[6]
Fe_3_O_4_-NG	4.1	− 53.6	4.68	[8]
Graphene/SiC	3.0	− 19.6	4.2	[9]
Ni@NG/NC	4.0	− 45.0	5.6	[11]
Fe_3_O_4_@C	2.0	− 20.6	4.8	[14]
RGO foam/Fe_2_O_3_	4.0	− 26.0	4.2	[15]
ERG/Si_3_N_4_	3.75	− 26.7	4.2	[17]
NGF	3.5	− 53.9	4.56	[19]
PAN/CNT/Fe_3_O_4_	1.5	− 59.85	4	[20]
RGO/PANI	3.5	− 36.9	4.2	[26]
NG-100	3.2	− 55.0	6.8	[27]
RGO/CNTs	2.75	− 55.0	3.5	[29]
CNWs/Si_3_N_4_	3.0	− 50.2	4.2	[35]
FeNi_3_/N-GN	1.45	− 57.2	3.4	[36]
Graphene/ZnO	2.2	− 45.1	2.2	[37]
CoNi@NG-NCP	2.5	− 24.0	4.3	[41]
BiFeO_3_/RGO	1.8	− 46.7	4.7	[43]
2VS_4_/rGO20-30%	1.4	− 48.4	2.3	This work
2VS_4_/rGO40-30%	1.4	− 37.4	4.8	This work

Comprehensive first-principles calculations are performed to probe the interfacial stabilities, interfacial bonding properties, and charge transfers at VS_4_/rGO heterointerface [44]. Specifically, the selections of crystal structure models and calculation parameters are shown in Figs. S14 and S15. VS_4_ can be grown on rGO along the (1 1 0) plane direction with the optimal lattice constant. The surface energy of VS_4_ (1 1 0)/rGO (1 0 0) can be calculated to about 0.798 eV, which is lower than VS_4_ (− 2 0 4)/rGO (1 0 0) of 6.657 eV and VS_4_ (0 2 0)/rGO (1 0 0) of 3.124 eV. Additionally, the V-terminated surface is more preferred to construct a heterointerface with rGO (1 0 0) than S-terminated surface (Fig S16). Therefore, the VS_4_/rGO heterointerface is prone to be bonded by V-C bands. As Fig. 6g shown, VS_4_ employs the (− 2 0 4), (0 2 0), and (1 1 0) lattice planes to match the plane of rGO (1 0 0), respectively. Figure 6g(I) demonstrates the charge density of VS_4_(− 2 0 4)/rGO (1 0 0) interface with an imaginary line on the heterointerface, and the C atoms above the heterointerface are far away from the bottom V atoms. Figure 6g(II) displays the charge density of VS_4_ (0 2 0)/rGO (1 0 0) interface, and the distance of V and C atoms is farther than the VS_4_ (− 2 0 4)/rGO (1 0 0) interface. Therefore, V and C atoms are extremely difficult to bond together. Figure 6g(III) exhibits the charge density of VS_4_ (1 1 0)/rGO (1 0 0) interface, and the shortest distance between V and C atoms is successfully realized. Due to the dislocation of the interface atom after relaxing, the V atoms are adjacent to C atoms with the formation of strong chemical bonds. Therefore, the VS_4_(1 1 0)/rGO (1 0 0) interface is easy to bond, and the result is consistent with the analysis of the above interface energy. Overall, the interfaces of VS_4_ (− 2 0 4)/rGO (1 0 0) and VS_4_ (1 1 0)/rGO (1 0 0) have great potential to form chemical bonds [45].

The chemical bands further require to be analyzed by charge density difference of heterointerface. The charge density differences of VS_4_ (− 2 0 4)/rGO (1 0 0) and VS_4_ (1 1 0)/rGO (1 0 0) heterointerfaces are shown in Fig. 6h. Figure 6h(I) shows that the charges are increasing between V and C atoms significantly, proving the formation of the covalent bonds between V and C atoms. Figure 6h(II) displays that the charges are increased near C atoms obviously, while the charges are decreased near V atoms considerably, indicating part of the charges of V atoms is transferred to C atoms. So, the ionic bonds have formed between the V and C atoms. The calculated results of charge density difference exactly match the electronic binding energy shift measured by XPS, further proving the electrons migrate from the VS_4_ to rGO (Fig. S6). Furthermore, the charge density difference in VS_4_/rGO heterointerface results in the enhancement of dipole and interfacial polarization relaxations, which greatly motivate microwave attenuation capability [46, 47].

**Fig. 7:**
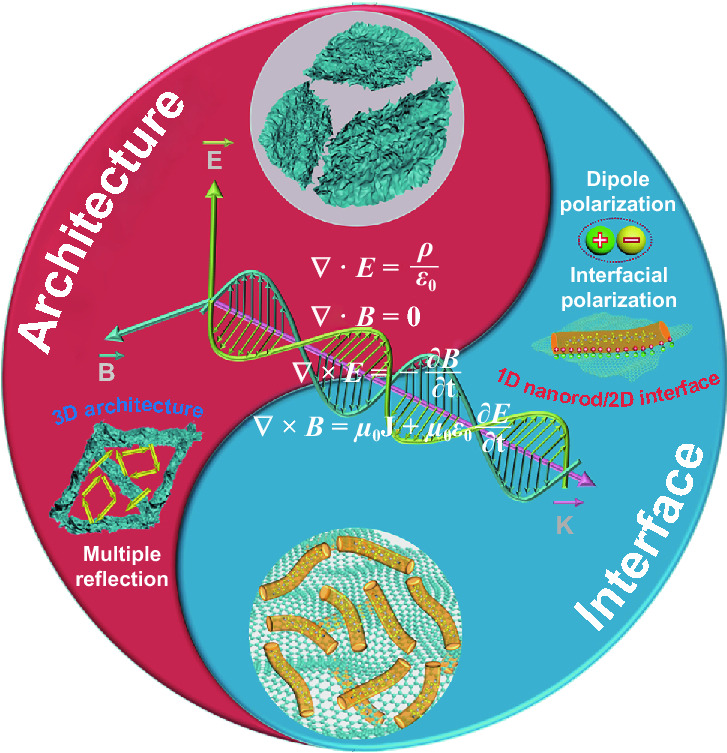
Schematic illustration for the microwave absorption mechanism of VS_4_/rGO heterostructure

Based on the abovementioned analysis, the strategies of architecture design and interface engineering open a new door for the development of enhanced and ultrathin microwave absorbents. Figure 7 shows the schematic illustration of microwave absorption mechanism for VS_4_/rGO heterostructure. Architecture design and interface engineering inspire the synergy of multi-dimensional advantages, including the anisotropy of 1D nanorods, 2D interface polarizations, and multiple microwave reflections or scatterings of 3D microarchitecture, facilitating to modulate impedance matching and attenuation constant [6, 10]. Firstly, 1D VS_4_ nanorods are anchored on rGO by van der Wall force and V-C bonds. The shape anisotropy and high aspect ratio of nanorods endow them with rapid charge transportation rate along the axial. The defects of VS_4_ nanorods and residual oxygen-containing functional groups of rGO break the balance of charge distributions in nanorods and interface [11, 26]. Eventually, the induced multitudinous dipoles and interfacial polarization centers significantly facilitate polarization relaxations [39, 48]. Secondly, the constructed 2D VS_4_/rGO heterointerfaces are joined together firmly. Numerous randomly distributed space charges accumulated at the heterointerface generate prominent dipole moments and logs. Therefore, the fine connections of VS_4_/rGO heterointerface effectively modulate the impedance matching and greatly improve the interfacial polarization relaxation [41]. Thirdly, the designed self-assembly 3D reticulum-like microporous VS_4_/rGO heterostructure provides a conductive paths for the electrons hopping and migrating between VS_4_ nanorods and rGO. The foamed conductive networks remarkably prolong the transmission paths of microwaves, which encourage the transformation of EM energy into heat energy by the induced microcurrent in alternating EM fields [42, 43]. In summary, the mixed-dimensional advantages originating from the synergy of architecture design and interface engineering can boost an appropriate balance of impedance matching and attenuation constant, eventually achieving high-performance microwave absorption materials.

## Conclusions

In summary, the strategies of architecture design and interface engineering are proposed to construct the self-assembly VS_4_/rGO heterostructure for the ultrathin and enhanced microwave absorbent. The synergy of microarchitecture and heterointerface can effectively modulate the impedance matching and attenuation constant of VS_4_/rGO heterostructure, thereby achieving enhanced microwave absorption performance at ultrathin thickness. The maximum RL of 2VS_4_/rGO40 heterostructure can reach as strong as − 43.5 dB at 14 GHz and 1.5 mm with the impedance matching and attenuation constant approaching 0.98 and 187, respectively. Furthermore, the EAB of 4.8 GHz can be achieved with an ultrathin thickness of 1.4 mm. DFT calculations provide a platform to decipher the interfacial polarization originating from heterointerface. Architecture design and interface engineering inspire multi-dimensional advantages, including anisotropic dipole polarization, interfacial polarization, and multiple reflections or scatterings of microwaves, eventually facilitating to achieve enhanced microwave absorption performance at ultrathin thickness. Therefore, architecture design and interface engineering offer an instructive attempt for the development of high-performance microwave absorption materials.

## Supplementary Information

Below is the link to the electronic supplementary material.

Supplementary file1 (PDF 3142 KB)

## References

[1] Shahzad F, Alhabeb M, Hatter CB, Anasori B, Hong SM (2016). Electromagnetic interference shielding with 2D transition metal carbides (MXenes). Science.

[2] Zhang Y, Huang Y, Zhang TF, Chang HC, Xiao PS (2015). Broadband and tunable high-performance microwave absorption of an ultralight and highly compressible graphene foam. Adv. Mater..

[3] Wu ZC, Pei K, Xing LS, Yu XF, You WB (2019). Enhanced microwave absorption performance from magnetic coupling of magnetic nanoparticles suspended within hierarchically tubular composite. Adv. Funct. Mater..

[4] Li Q, Zhang Z, Qi LP, Liao QL, Kang Z (2019). Toward the application of high frequency electromagnetic wave absorption by carbon nanostructures. Adv. Sci..

[5] Zhao HH, Wang FY, Cui LR, Xu XZ, Han XJ (2021). Composition optimization and microstructure design in MOFs-derived magnetic carbon-based microwave absorbers: a review. Nano-Micro Lett..

[6] Liu Y, Chen Z, Zhang Y, Feng R, Chen X (2018). Broadband and lightweight microwave absorber constructed by in situ growth of hierarchical CoFe_2_O_4_/reduced graphene oxide porous nanocomposites. ACS Appl. Mater. Interfaces.

[7] Liu QH, Cao Q, Bi H, Liang CY, Yuan KP (2015). CoNi@SiO_2_@TiO_2_ and CoNi@Air@TiO_2_ microspheres with strong wideband microwave absorption. Adv. Mater..

[8] He P, Cao MS, Cao WQ, Yuan J (2021). Developing MXenes from wireless communication to electromagnetic attenuation. Nano-Micro Lett..

[9] Liang LY, Li QM, Yan X, Feng YZ, Wang YM (2021). Multifunctional magnetic Ti_3_C_2_Tx MXene/graphene aerogel with superior electromagnetic wave absorption performance. ACS Nano.

[10] Li Q, Zhang Z, Xun XC, Gao FF, Zhao X (2020). Synergistic engineering of dielectric and magnetic losses in M-Co/RGO nanocomposites for use in high-performance microwave absorption. Mater. Chem. Front..

[11] Yuan HR, Yan F, Li CY, Zhu CL, Zhang XT (2018). Nickel nanoparticle encapsulated in few-layer nitrogen-doped graphene supported by nitrogen-doped graphite sheets as a high-performance electromagnetic wave absorbing material. ACS Appl. Mater. Interfaces.

[12] Liu PB, Gao S, Zhang GZ, Huang Y, You WB (2021). Hollow engineering to Co@N-doped carbon nanocages via synergistic protecting-etching strategy for ultrahigh microwave absorption. Adv. Funct. Mater..

[13] Hou JH, Inganäs O, Friend RH, Gao F (2018). Organic solar cells based on non-fullerene acceptors. Nat. Mater..

[14] Du YC, Liu WW, Qiang R, Wang Y, Han XJ (2014). Shell thickness-dependent microwave absorption of core–shell Fe_3_O_4_@C composites. ACS Appl. Mater. Interfaces.

[15] Zhang H, Xie A, Wang C, Wang H, Shen Y (2013). Novel rGO/α-Fe_2_O_3_ composite hydrogel: synthesis, characterization and high performance of electromagnetic wave absorption. J. Mater. Chem. A.

[16] Gao S, Zhang GZ, Wang Y, Han XP, Huang Y (2021). MOFs derived magnetic porous carbon microspheres constructed by core-shell Ni@C with high-performance microwave absorption. J. Mater. Sci. Technol..

[17] Ye F, Song Q, Zhang ZC, Li W, Zhang SY (2018). Direct growth of edge-rich graphene with tunable dielectric properties in porous Si_3_N_4_ ceramic for broadband high-performance microwave absorption. Adv. Funct. Mater..

[18] Zhao Y, Zuo X, Guo Y, Huang H, Zhang H (2021). Structural engineering of hierarchical aerogels comprised of multi-dimensional gradient carbon nanoarchitectures for highly efficient microwave absorption. Nano-Micro Lett..

[19] Liu PB, Zhang YQ, Yan J, Huang Y, Xia L (2019). Synthesis of lightweight N-doped graphene foams with open reticular structure for high-efficiency electromagnetic wave absorption. Chem. Eng. J..

[20] Li Y, Liu XF, Nie XY, Yang WW, Wang YD (2018). Multifunctional organic–inorganic hybrid aerogel for self-cleaning, heat-insulating, and highly efficient microwave absorbing material. Adv. Funct. Mater..

[21] L.P. Wu, K.M, Zhang, J.Y. Shi, F. Wu, X.F. Zhu et al., Metal/nitrogen co-doped hollow carbon nanorods derived from self-assembly organic nanostructure for wide bandwidth electromagnetic wave absorption. Compos. Part B Eng.; **228**:3 109424 (2022). 10.1016/j.compositesb.2021.109424

[22] Chen C, Xi JB, Zhou EZ, Peng L, Chen ZC (2018). Porous graphene microflowers for high-performance microwave absorption. Nano-Micro Lett..

[23] Lv HL, Guo YH, Wu G, Ji GB, Zhao Y (2017). Interface polarization strategy to solve electromagnetic wave interference issue. ACS Appl. Mater. Interfaces.

[24] Xie AM, Sun MX, Zhang K, Jiang WC, Wu F (2016). In situ growth of MoS_2_ nanosheets on reduced graphene oxide (RGO) surfaces: interfacial enhancement of absorbing performance against electromagnetic pollution. Phys. Chem. Chem. Phys..

[25] Rout CS, Kim BH, Xu XD, Yang J, Jeong HY (2013). Synthesis and characterization of patronite form of vanadium sulfide on graphitic layer. J. Am. Chem. Soc..

[26] X.G. Chen, F.C. Meng, Z.W. Zhou, X. Tian, L.M Shan et al., One-step synthesis of graphene/polyaniline hybrids by in situ intercalation polymerization and their electromagnetic properties. Nanoscale **6**(14), 8140–8148 (2014). 10.1039/C4NR01738B 10.1039/c4nr01738b 24922345

[27] Ning MQ, Kuang BY, Wang L, Li JB, Jin HB (2021). Correlating the gradient nitrogen doping and electromagnetic wave absorption of graphene at gigahertz. J. Alloys Compd..

[28] Xu XD, Jeong S, Rout CS, Oh P, Ko M (2014). Lithium reaction mechanism and high-rate capability of VS_4_–graphene nanocomposite as an anode material for lithium batteries. J. Mater. Chem. A.

[29] Kong L, Yin XW, Yuan XY, Zhang YJ, Liu XM (2014). Electromagnetic wave absorption properties of graphene modified with carbon nanotube/poly (dimethyl siloxane) composites. Carbon.

[30] Cheng SY, Xie AM, Pan XH, Zhang KX, Zhang C (2021). Modulating surficial oxygen vacancy of the VO_2_ nanostructure to boost its electromagnetic absorption performance. J. Mater. Chem. C.

[31] Sun H, Che RC, You X, Jiang YS, Yang ZB (2014). Cross-stacking aligned carbon-nanotube films to tune microwave absorption frequencies and increase absorption intensities. Adv. Mater..

[32] Pang Q, Wei YJ, Yu YH, Bian XF, Wang XD (2018). VS_4_ nanoparticles anchored on graphene sheets as a high-rate and stable electrode material for sodium ion batteries. Chemsuschem.

[33] Wang SZ, Gong F, Yang SZ, Liao JX, Wu MQ (2018). Graphene oxide-template controlled cuboid-shaped high-capacity VS_4_ nanoparticles as anode for sodium-ion batteries. Adv. Funct. Mater..

[34] Zhou YL, Tian J, Xu HY, Yang J, Qian YT (2017). VS_4_ nanoparticles rooted by a-C coated MWCNTs as an advanced anode material in lithium-ion batteries. Energy Storage Mater..

[35] Pan HX, Yin XW, Xue JM, Cheng LF, Zhang LT (2016). In-situ synthesis of hierarchically porous and polycrystalline carbon nanowires with excellent microwave absorption performance. Carbon.

[36] Feng J, Zong Y, Sun Y, Zhang Y, Yang X (2018). Optimization of porous FeNi_3_/N-GN composites with superior microwave absorption performance. Chem. Eng. J..

[37] Han MK, Yin XW, Kong L, Li M, Duan WY (2014). Graphene-wrapped ZnO hollow spheres with enhanced electromagnetic wave absorption properties. J. Mater. Chem..

[38] Pan F, Liu ZC, Deng BW, Dong YY, Zhu XJ (2021). Lotus leaf-derived gradient hierarchical porous C/MoS_2_ morphology genetic composites with wideband and tunable electromagnetic absorption performance. Nano-Micro Lett..

[39] Zhao Y, Hao LL, Zhang XD, Tan SJ, Li HH (2021). A novel strategy in electromagnetic wave absorbing and shielding materials design: multi-responsive field effect. Small Sci..

[40] Zhang X, Qiao J, Jiang YY, Wang FL, Tian XL (2021). Carbon-based MOF derivatives: emerging efficient electromagnetic wave absorption agents. Nano-Micro Lett..

[41] Zhang X, Yan F, Zhang S, Yuan HR, Zhu CL (2018). Hollow N-doped carbon polyhedron containing CoNi alloy nanoparticles embedded within few-layer N-doped graphene as high-performance electromagnetic wave absorbing material. ACS Appl. Mater. Interfaces.

[42] Wang JQ, Liu L, Jiao SL, Ma KJ, Lv J (2020). Hierarchical carbon fiber@MXene@MoS_2_ core-sheath synergistic microstructure for tunable and efficient microwave absorption. Adv. Funct. Mater..

[43] Gao X, Wang Y, Wang QG, Wu XM, Zhang WZ (2019). Facile synthesis of a novel flower-like BiFeO_3_ microspheres/graphene with superior electromagnetic wave absorption performances. Ceram. Int..

[44] Yu S, Ran SJ, Zhu H, Eshun K, Shi C (2018). Study of interfacial strain at the α-Al_2_O_3_/monolayer MoS_2_ interface by first principle calculations. Appl. Surf. Sci..

[45] Liu XJ, Yin YJ, Ren Y, Wei H (2014). The investigation of the C-Si interface structure in diamond/Si nano-composite films with first principle method. Appl. Surf. Sci..

[46] Guo XY, Zhang Y, Jung YG, Li L, Knapp J (2016). Ideal tensile strength and shear strength of ZrO_2_(111)/Ni (111) ceramic-metal Interface: a first principle study. Mater. Design.

[47] Wang F, Gu WH, Chen JB, Huang QQ, Han MY (2022). Improved electromagnetic dissipation of Fe doping LaCoO_3_ toward broadband microwave absorption. J. Mater. Sci. Technol..

[48] Wang F, Gu WH, Chen JB, Wu Y, Zhou M (2021). The point defect and electronic structure of K doped LaCo_0.9_Fe_0.1_O_3_ perovskite with enhanced microwave absorbing ability. Nano Res..

