# Supplementary material for: Architecture Design and Interface Engineering of Self-assembly VS4/rGO Heterostructures for Ultrathin Absorbent
Source: Nanomicro Lett. 2022 Feb 25;14:67. doi: 10.1007/s40820-022-00809-5 (PMC8873340; doi:10.1007/s40820-022-00809-5)
Supplement: Supplementary file 1 — Supplementary file1 (PDF 3142 KB) [file 40820_2022_809_MOESM1_ESM.pdf]

Supporting Information for

Architecture Design and Interface Engineering of Self-Assembly VS_4/rGO Heterostructures for Ultrathin Absorbent

Qi Li[1,#], Xuan Zhao[1,#], Zheng Zhang[1], Xiaochen Xun[1], Bin Zhao[1], Liangxu Xu[1], Zhuo Kang[1], Qingliang Liao[1,*], Yue Zhang[1,*]

[1]Academy for Advanced Interdisciplinary Science and Technology, Beijing Advanced Innovation Center for Materials Genome Engineering, Beijing Key Laboratory for Advanced Energy Materials and Technologies, School of Materials Science and Engineering, University of Science and Technology Beijing, Beijing 100083, People's Republic of China

[#]Qi Li and Xuan Zhao have contributed equally to this work

*Corresponding authors. E-mail: liao@ustb.edu.cn (Qingliang Liao), yuezhang@ustb.edu.cn (Yue Zhang)

S1 Chemical materials

Sodium orthovanadate (Na_3VO_4, AR, 99%) and thioacetamide (AR, 98%) were purchased from Shanghai Macklin Biochemical Co., Ltd. Sodium hydroxide was purchased from Aladdin In. Co. Ethanol (AR, 99.7%) was obtained by Sinopharm Chemical Reagent Co., Ltd. The deionized water was prepared by laboratory water purification system. The above reagents were analytical and used without further purification.

S2 Supplementary Figures and Tables

Table S1 The prepared VS_4/rGO heterostructures with different content of VS_4

Chemical reagent	2VS_4/rGO20	2VS_4/rGO40	2VS_4/rGO60	1VS_4/rGO40	3VS_4/rGO40
Na_3VO_4 (mmol)	5	5	5	2.5	10
CH_3CSNH_2 (mmol)	25	25	25	12.5	50
GO (mg)	20	40	60	40	40

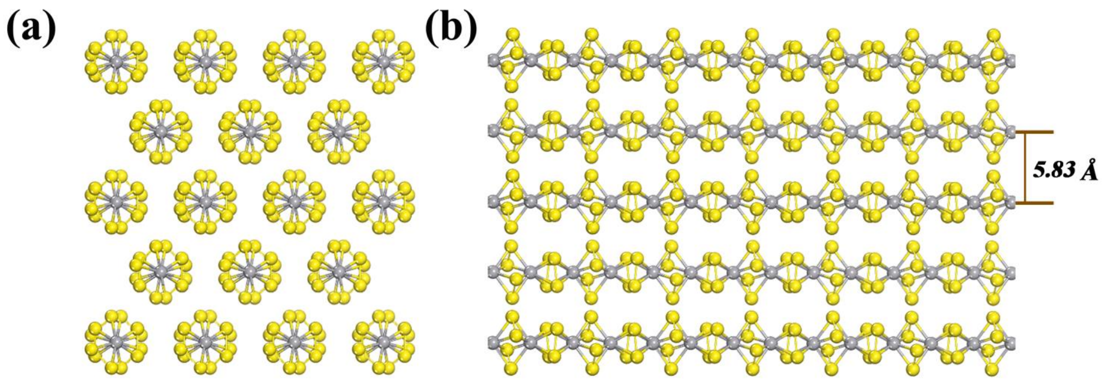

Fig. S1 The crystal structure schematic of VS_4: **a** top view of repeating unit of 1D chain structure, **b** side view image of monoclinic VS_4 (the yellow ball represents the S atoms, and the gray ball represents the V atoms)

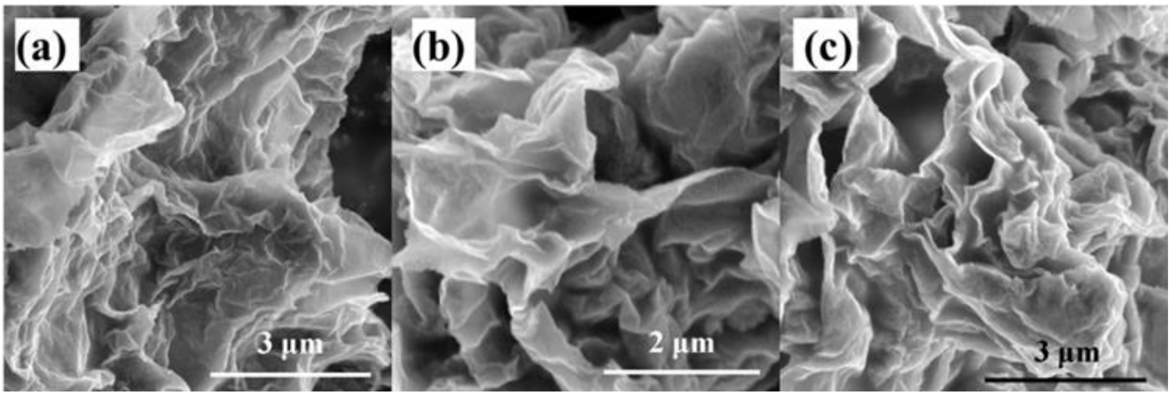

Fig. S2 SEM images of different VS_4/rGO heterostructures: **a** $2VS_4$/rGO20, **b** $2VS_4$/rGO40 and **c** $2VS_4$/rGO60

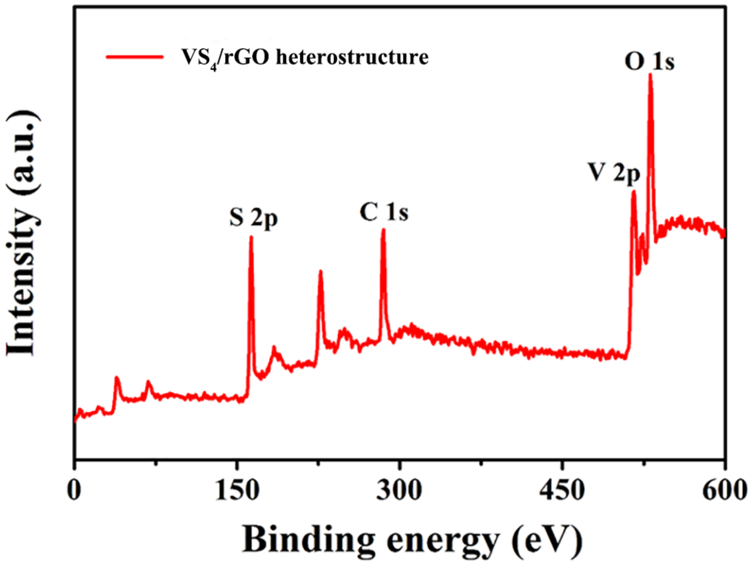

Fig. S3 XPS spectrum of VS_4/rGO heterostructure

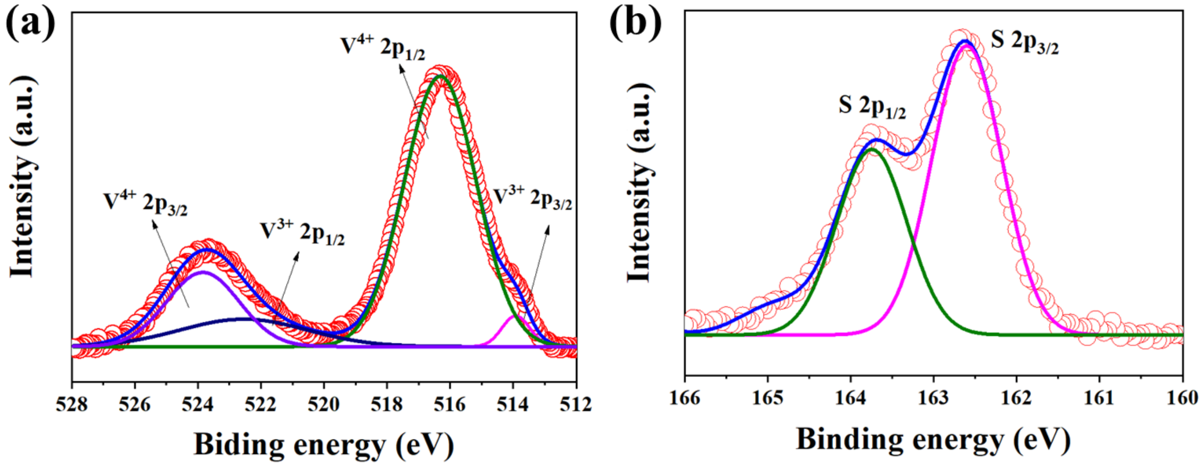

Fig. S4 High-resolution of XPS spectra for VS_4 nanorods: **a** V 2p, **b** S 2p

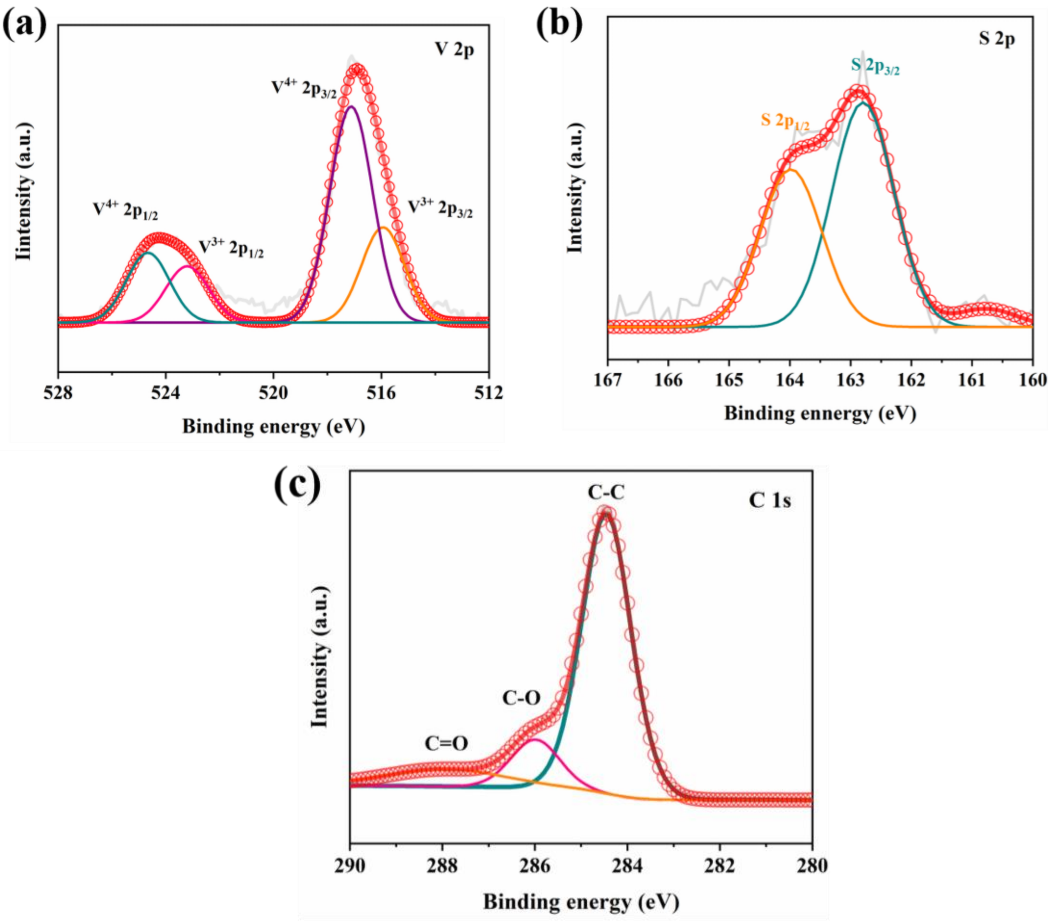

Fig. S5 High-resolution of XPS spectra for VS_4/rGO nanocomposite: **a** V 2p, **b** S 2p, and **c** C 1s

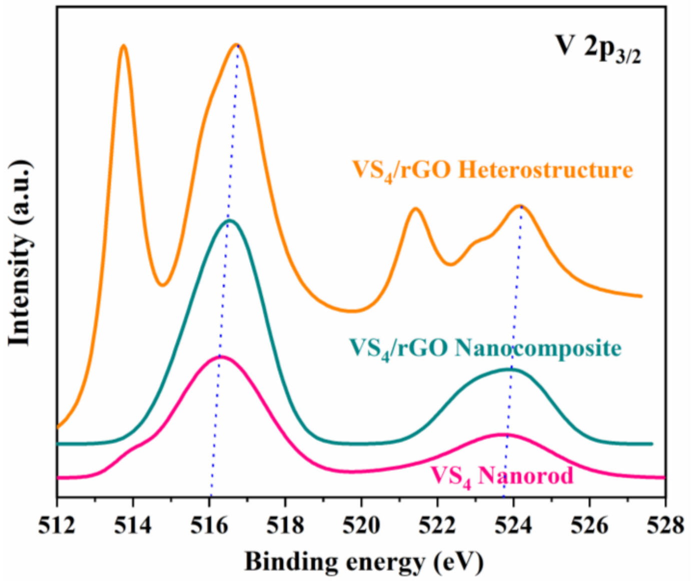

Fig. S6 High-resolution of V 2p spectra for VS_4/rGO heterostructure, VS_4/rGO nanocomposite and VS_4 nanorod

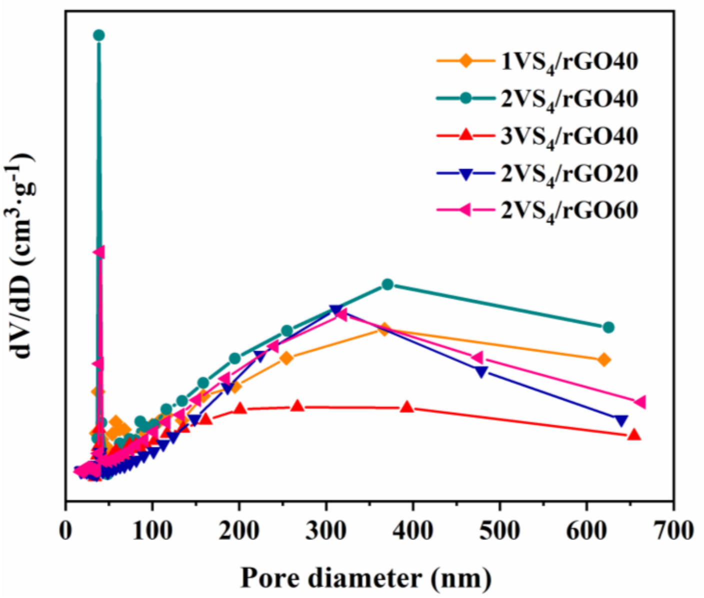

Fig. S7 Pore size distributions of VS_4/rGO heterostructures

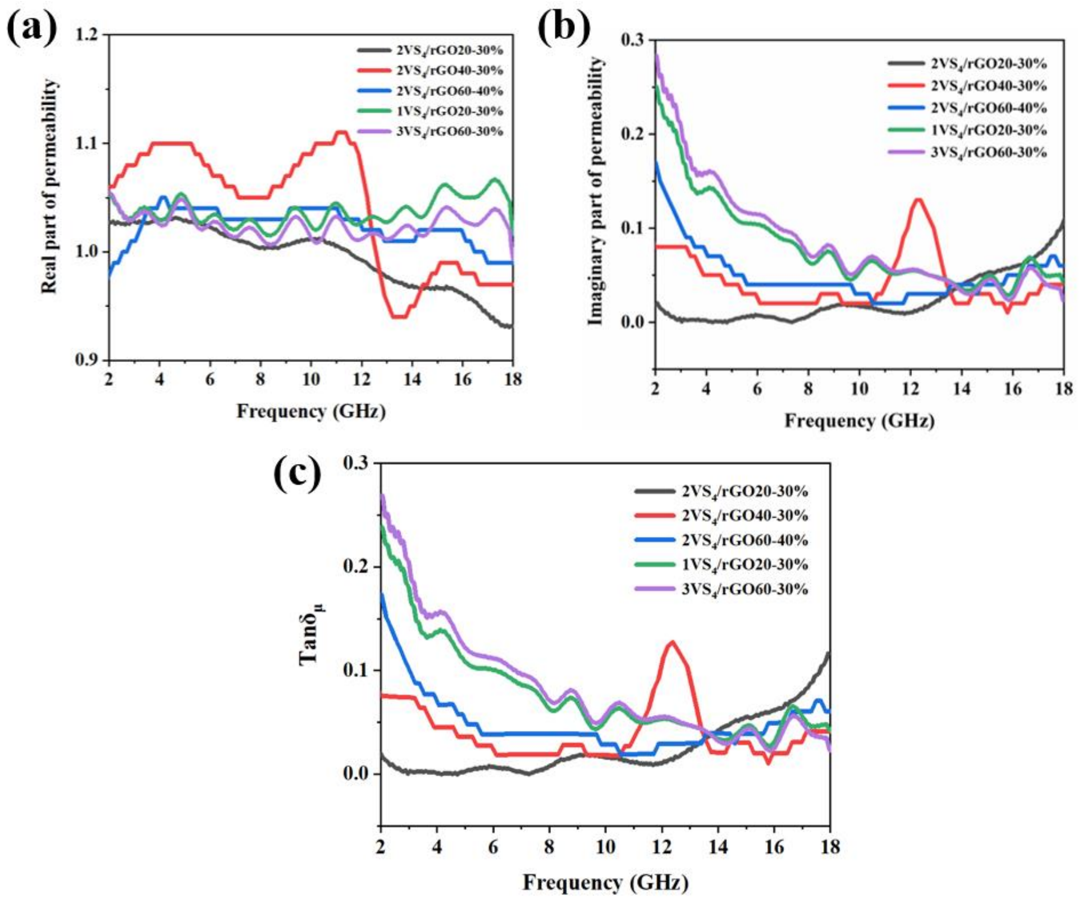

Fig. S8 Permeability and magnetic loss tangent of VS_4/rGO heterostructures in the frequency range of 2-18 GHz: **a** μ', **b** μ'', **c** Tan δ_μ

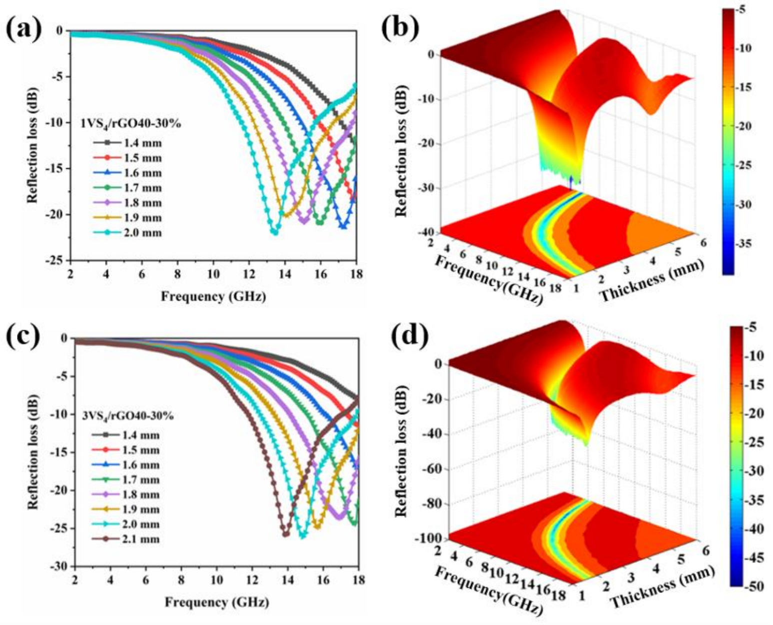

Fig. S9 RL curves and 3D presentations at the thicknesses of 1 to 6 mm in the frequency range of 2-18 GHz: **a, b** 1VS_4/rGO40-30%, **c, d** 3VS_4/rGO40-30%

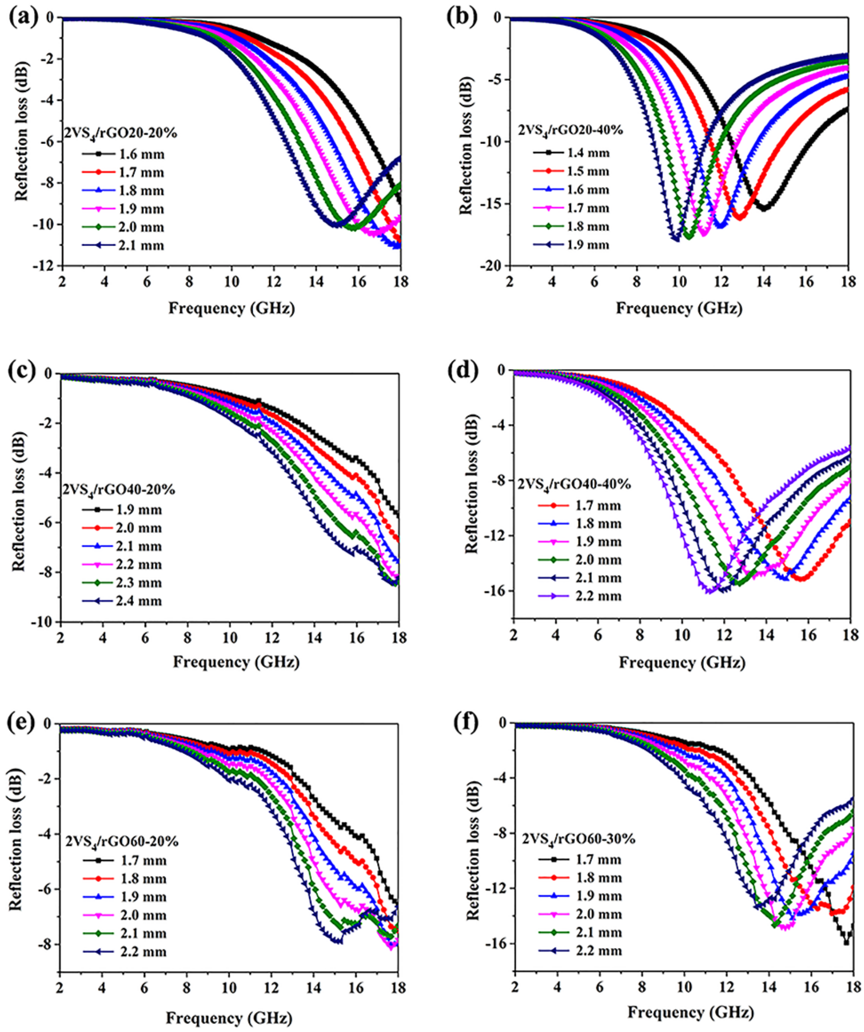

Fig. S10 RL curves of VS_4/rGO heterostructures at the thicknesses of 1 to 6 mm in the frequency range of 2-18 GHz: **a** 2VS_4/rGO20-20%, **b** 2VS_4/rGO20-40%, **c** 2VS_4/rGO40-20%, **d** 2VS_4/rGO40-40%, **e** 2VS_4/rGO60-20%, and **f** 2VS_4/rGO60-30%

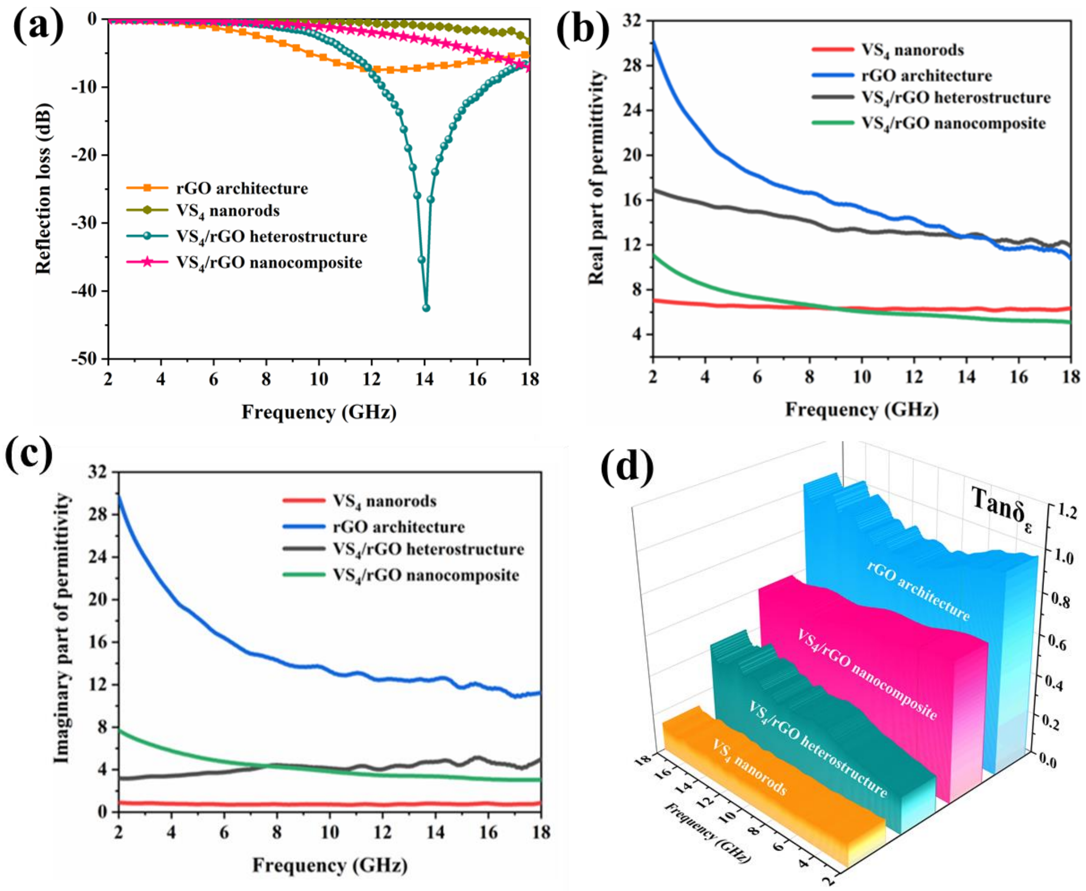

Fig. S11 Permittivity and dielectric loss tangent of rGO, VS_4 nanorods, VS_4/rGO heterostructure, and VS_4/rGO nanocomposite in the frequency of 2-18 GHz: **a** real part of permittivity, **b** imaginary part of permittivity. **c** Tanδ_ε. **d** RL curves of rGO, VS_4 nanorods, VS_4/rGO heterostructure, and VS_4/rGO nanocomposite with a thickness of 1.5 mm

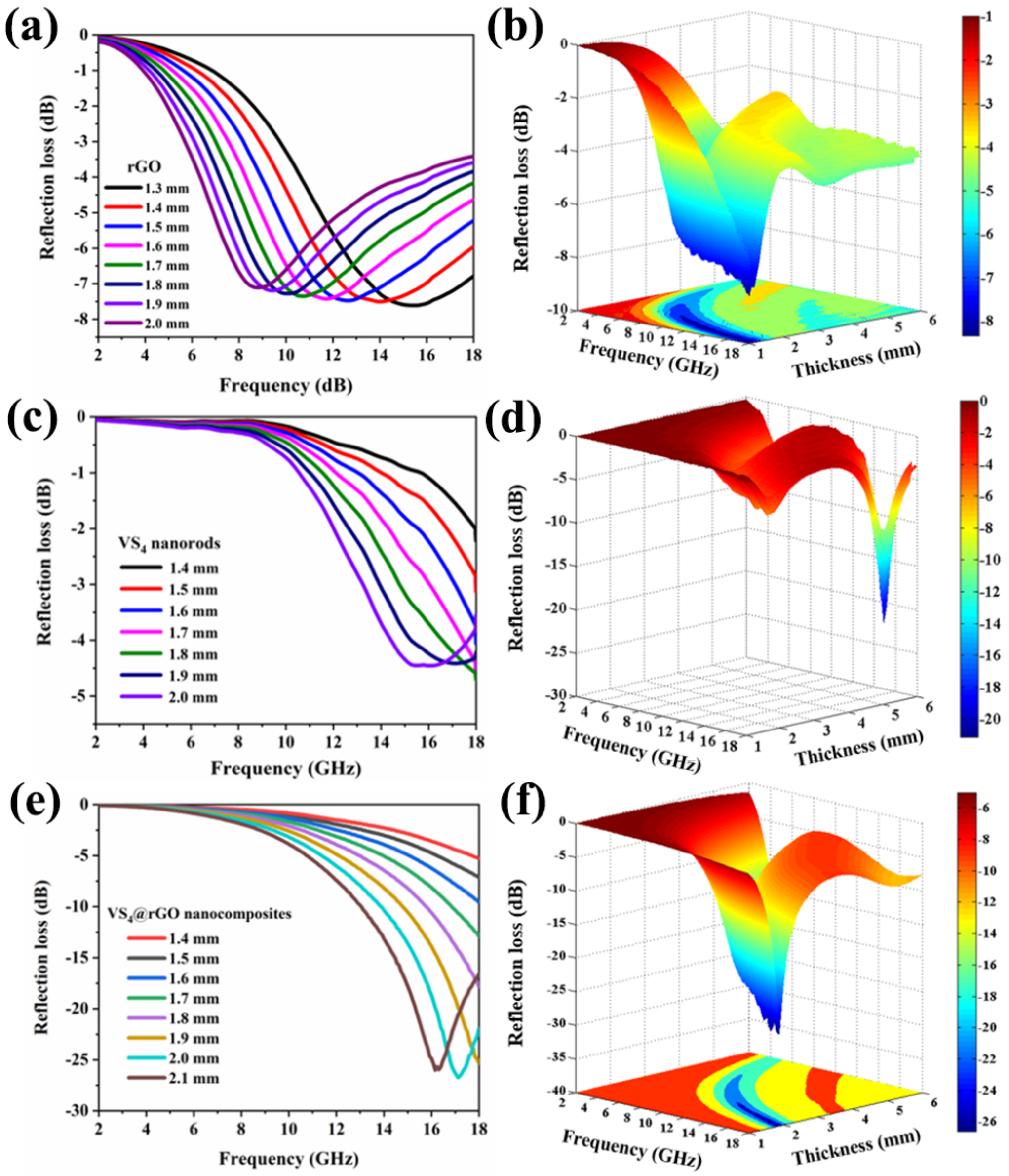

Fig. S12 RL curves and 3D presentations at the thicknesses of 1 to 6 mm in the frequency range of 2-18 GHz: **a** and **b** rGO, **c** and **d** VS_4 nanorods, **e** and **f** VS_4/rGO nanocomposite

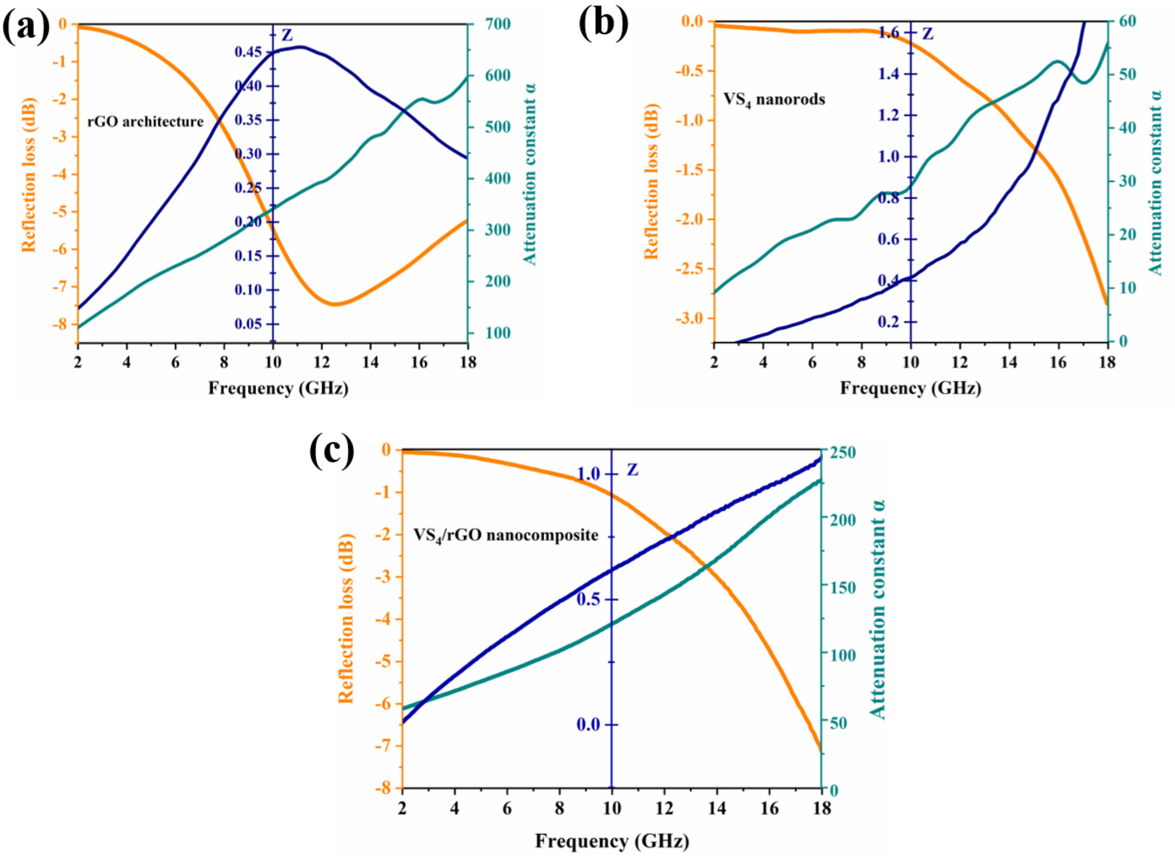

Fig. S13 Frequency dependence of RL, attenuation constant α and the modulus of normalized input impedance ($|Z_{in}/Z_0|$) with a thickness of 1.5 mm for **a** rGO architecture, **b** VS_4 nanorods, and **c** VS_4/rGO nanocomposite

S3 DFT Calculation

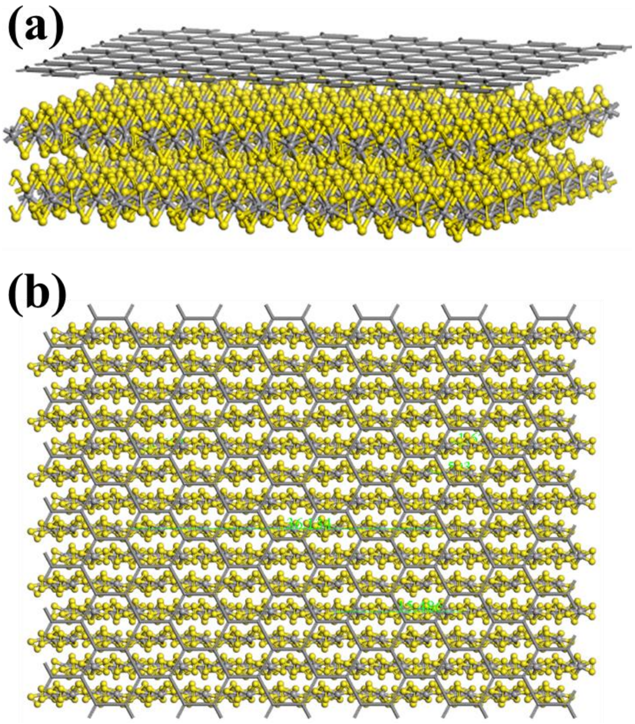

Fig. S14 The crystal structure of VS_4/rGO heterostructure

The crystal structures of VS_4 and VS_4/rGO heterostructure are shown in Fig. S14, respectively. The lattice constants of VS_4 are *a=6.775 Å, b=10.420 Å, c=12.110 Å and α=γ=90°, β=100.8°*. Each parameter needs to be specified to generate input files when calculated. Therefore, some parameters must be selected carefully, such as energy cut-off and k-points in Brillouin zone during the calculation. The larger values of the energy cut-off and k-points, the more accurate calculation results can be achieved. However, the larger values of parameter not only increase the difficulties of calculation, but also completely unnecessary. Therefore, the optimized structure and model can be achieved by employing the smaller parameters that are enough to ensure their accuracies.

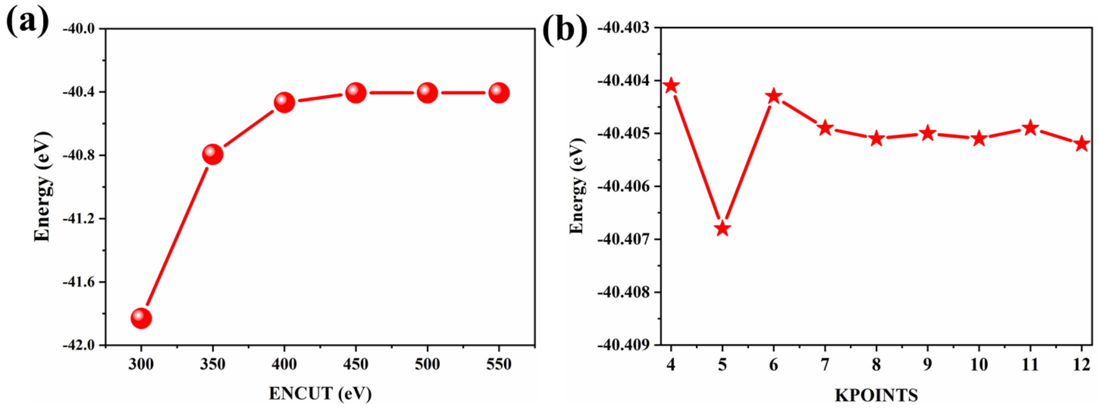

Fig. S15 Determination of energy cut-off (ENCUT) and K-mesh of VS_4: **a** ENCUT-Energy, **b** K mesh-Energy

The ENCUT-Energy of VS_4 is shown in Fig. 14a. It can be seen that the curve tends to be gentle with 0.001eV/atom after the ENCUT value reaches 400 eV. Therefore, the optimal energy cut-off is about 450 eV. As Fig. 14b shown, when the K mesh is chosed as 8×5×4, the energy is convergent and the curve becomes gentle as well. So, the optimal K mesh is obtained as 8×5×4. The formation enthalpy of bulk VS_4 is as follows Equation (1): [S1]

$$\Delta_r H_m(M_xN_y)=\frac{E_{total}\left(M_xN_y\text{-}xE_{bulk}(M)\text{-}yE_{bulk}(N)\right)}{n} \tag{1}$$

Where $E_{total}(M_xN_y)$ is the total cell energy; $E_{bulk}(M)$ and $E_{bulk}(N)$ are the chemical potential of *M* or *N* atom in the bulk state respectively; *n* is the total number of formula per cell. The formation enthalpy of VS_4 is calculated as -1.26eV, which is similar to the experimental value (-0.98 eV) [S2].

It's worthwhile mentioning that three types of VS_4 surface structures are selected to match graphene (100). The three surface structures are polar interfaces, whose surface energy can be calculated by the following Equation (2). [S3]

$$\sigma=\frac{1}{2A}(E_{slab}\text{-}N_V\mu_V^{slab}\text{-}N_S\mu_S^{slab}+PV\text{-}TS) \tag{2}$$

Where A is the surface area of the surface model; Eslab is the system energy of the calculated surface model; NV is the atomic number of V; NS is the atomic number of S; μ_V^{slab} is the chemical potential of V atom; μ_S^{slab} is the chemical potential of S atom. Generally, when the surface structure is fully relaxed, it is in equilibrium with the bulk phase. Therefore, the chemical potential of the surface structure is about the same as the bulk phase. As the following Equation (3) shown:

$$\mu_{VS_4}^{slab}=\mu_{VS_4}^{bulk}=\mu_V^{slab}+2\mu_S^{slab} \tag{3}$$

Where $\mu_{VS_4}^{bulk}$ is the system energy of the bulk VS4 after optimized.

Combining Equation (2) and (3), it can be obtained as follows Equation (4):

$$\sigma=\frac{1}{2A}(E_{slab}-N_V\mu_V^{bulk}+(4N_V-N_S)\mu_S^{slab} \tag{4}$$

In order to simplify the calculation, it can be approximated as $\mu_S^{slab}\approx\mu_S^{bulk}$. Then these values are put into Equation (4), the surface energies of VS4(-2 0 4)/rGO(1 0 0), VS4(0 2 0)/rGO(1 0 0) and VS4(1 1 0)/rGO(1 0 0) are about 3.124 eV, 6.657 eV, 0.798 eV, respectively.

The slabs values of the three surface energies of VS4(-2 0 4)/rGO(1 0 0), VS4(0 2 0)/rGO(1 0 0) and VS4(1 1 0)/rGO(1 0 0) are put into Equation (4). The Equations can be obtained as follows:

$$\sigma_{V\text{-}terminated\text{-}VS_4}=\frac{1}{2A}(E_{slab}-24\mu_{VS_4}^{bulk}+8\mu_S^{slab}) \tag{5}$$

$$\sigma_{S\text{-}terminated\text{-}VS_4}=\frac{1}{2A}(E_{slab}+24\mu_{VS_4}^{bulk}+8\mu_S^{slab}) \tag{6}$$

In fact, the chemical potential of each atom in the surface structures is slightly smaller than that in the bulk. So it can be obtained as follows:

$$\Delta\mu_S\leq\mu_S^{slab}-\mu_S^{bulk}\leq 0 \tag{7}$$

The formation enthalpy of bulk is given in Equation (8):

$$\mu_{VS_4}^{bulk}=\mu_V^{bulk}+\mu_S^{bulk}+\Delta H_{VS_4} \tag{8}$$

Combining the Equation (5)- (8), it can be obtained as follows:

$$\frac{1}{4}\Delta H_{VS_4}\leq\mu_S^{slab}-\mu_S^{bulk}\leq 0 \tag{9}$$

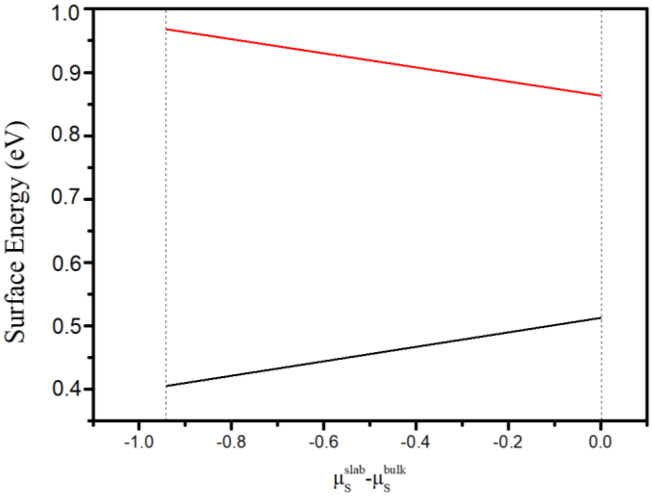

Fig. S16 Surface energy fluctuation

Based on the above calculation, the relation curves of the surface energy are changed ($\mu_S^{slab}-\mu_S^{bulk}$) with the chemical potential fluctuation of VS_4 (1 1 0) as is shown in Fig. 15. and the other surface energy fluctuation curves are similar to VS_4(1 1 0). The red and black curves represent the V-terminated and S-terminated surfaces, respectively. The V-terminated surface was selected to construct interface structures with Graphene(100). The energy values of the two terminations are quite different, but the change is not significant in the range of its fluctuation.

Supplementary References

[S1] C.C. Zhao, Y.F. Zhou, X.L. Xing, S. Liu, X.J. Ren et al., Investigation on the relationship between NbC and wear-resistance of Fe matrix composite coatings with different C contents. Appl. Surface Sci. **439**, 468-474 (2018). https://doi.org/10.1016/j.apsusc.2018.01.034

[S2] C.S. Rout, B.H. Kim, X.D. Xu, J. Yang, H.Y. Jeong, et al., Synthesis and characterization

of patronite form of vanadium sulfide on graphitic layer. J. Am. Chem. Soc. **135**(23), 8720-8725 (2013). https://doi.org/10.1021/ja403232d

[S3] J.B. Wang, J. Yang, C.X. Wang, Y.F. Zhou, X.L. Xing et al., First-principles calculation on $LaAlO_3$ as the heterogeneous nucleus of TiC. Comput. Mater. Sci. **101**, 108-114 (2015). https://doi.org/10.1016/j.commatsci.2015.01.024
